# Book of Abstracts

**DOI:** 10.1080/24740527.2024.2366131

**Published:** 2024-10-30

**Plenary: Eliminating Anti-Indigenous Racism & Increasing the Cultural Safety of the Health System**

Nel Wieman

Mishi-baawitigong First Nation, Anishinaabe Nation, Adopted member of Takaya Wolf Clan, FNHA family

Anti-Indigenous racism has been documented as widespread in BC’s health system, and it plays a significant role in Indigenous patients accessing and receiving care for health conditions, including chronic pain and substance use. Clinicians, researchers, and those in other health-related fields will benefit from learning how to develop cultural humility in their work that contributes to increased cultural safety. Trauma, historical and contemporary, is related to pain and addiction and learning how this impacts Indigenous patients is critical to increasing cultural safety. The BC Cultural Safety and Humility Standard is the first of its kind in Canada, and plans are underway to create a national standard. Tools such as these can assist clinicians, researchers, and others to develop a “two-eyed seeing” approach that will result in improved health outcomes for Indigenous people in Canada.

**At the end of this session, participants will be able to**:

- Describe what antiracism, cultural safety and cultural humility look like in their individual settings (clinical, research, etc.).
- Identify how trauma is a major factor in pain & substance use for Indigenous (FN/M/I) people.
- Integrate a “two-eyed seeing” approach into their individual settings (clinical, research, etc.).

**Plenary: The Mechanistic Mélange of Pain**

Michael Salter

Program in Neuroscience & Mental Health, Hospital for Sick Children, Dept. of Physiology, University of Toronto

Advancing new therapeutic approaches remains a significant challenge in the field of pain. Transcriptomic analysis of changes in gene expression in the spinal cord dorsal horn have led to cataloging diverse cellular alterations in response to peripheral nerve injury but have focused on phenomenology and classifying transcriptomic changes. Recently, we took a purposeful approach of exploring the possibility of identifying pain-relieving drugs by simultaneously looking not just between sexes in a single species but between sexes in two species. Surprisingly, given the large emphasis on sex differences across biomedical sciences we found that there are many more commonalities than differences between sexes and across species in the gene expression changes produced in the spinal dorsal horn. To take our analysis forward we developed a way to use this information to make testable predictions about drug response. We used pathway analysis to define the molecular network of proteins encoded by the genes in which transcript expression had changed. Because of the power of our four-way analysis we not only identified known pathways – which validated our analyses – but we discovered connections and hubs not seen previously. With this information we developed a workflow through mining the database of FDA-approved drugs and interrogating this with the common nodes and hubs we had identified. The top hit from this analysis was fostamatinib, the molecular target of which is the nonreceptor tyrosine kinase SYK, which our analysis had identified as a key node in the interactome. administrating the active two structurally distinct SYK inhibitors reversed pain hypersensitivity and, as our analysis predicted, did this in both sexes. Thus, we identified and showed the efficacy of agents that could not have been previously predicted to have analgesic properties.

**At the end of this session, the participants will be able to**:

- Recognize the diverse mechanisms for pain sensitization.
- Identify how gene expression analysis can be used to identify new molecular targets for pain-reducing therapies.
- Outline sex differences and sex similarities in pain mechanisms.

**Concurrent Session One**

**Exploring Placebo and Nocebo Effects in Pain Modulation: From Rodents to Humans**

**Session Chair:** Jeffrey Mogil

McGill University

**Session Abstract:** The placebo and nocebo effects represent two of the most striking and well-known demonstrations of the brain’s influence on the experience of pain. There is growing interest in understanding placebo and nocebo modulation of pain from basic science and clinical perspectives. There is also a strong push from the basic science community to reveal deeper mechanistic insight into how prior experiences can shape pain perception – both positively and negatively. This includes moving beyond a narrow conceptualization of the placebo/nocebo effects as only expectancy-driven and considering other aspects such as conditioning, contextual influences, and social learning. Conditioning, often through repeated associations, can influence pain modulation, and understanding these mechanisms opens new avenues for therapeutic intervention. The symposium will also explore the role of social learning in placebo and nocebo effects and a shift in our understanding of these phenomena. Recent research demonstrates that placebo/nocebo effects can be learned by observing others experiencing harm, not just through individual experiences. This highlights the significance of social learning in influencing pain perception and broadens our perspective on the origins of placebo and nocebo responses. Corder will present unpublished findings on the malleable nature of pain perception in preclinical pain contexts, highlighting vlPAG microcircuits for opioidergic signaling under probabilistic need-states. Martin will illustrate novel mouse models to study nocebo hyperalgesia and their use in understanding the brain circuits and neuropeptides that regulate social and contextual nocebo responses. Finally, Colloca will discuss recent advancements in understanding nocebo effects in humans, shedding light on clinical and ethical considerations.

**At the end of this session, participants will be able to**:

- Describe placebo/nocebo pain responses in rodents.
- Identify neurobiological circuits related to placebo/nocebo pain modulation.
- Define nocebo effects from a clinical and ethical perspective.

**Speaker One**

**Endogenous Calibration of Nociceptive Dynamics in Periaqueductal Gray Opioidergic Circuits**

Gregory Corder

University of Pennsylvania

Opioid analgesics and endogenous peptides engage mu-opioid receptors (MOR) signaling across multiple brain regions to alleviate pain. Notably, the ventrolateral periaqueductal gray (vlPAG) plays a dual functional role for both nociceptive processing and robust antinociception. However, the molecular identity, signaling dynamics, and plasticity of MOR+ neurons in vlPAG and their role in pain and endogenous analgesia remains unclear. In this talk, Dr. Corder will discuss recent studies conducted by his lab that characterized nociceptive MOR+ neural populations in the vlPAG to gain insight into the molecular markers and temporal dynamics that define this functional ensemble. To assess endogenous opioid analgesia, a novel nonpharmacological placebo model that utilizes instrumental conditioning to drive expectation-mediated analgesia was developed. In our optimized endogenous analgesia conditioning (EAC) paradigm, EAC mice prefer to spend significantly more time in the formerly innocuous paired context and display attenuated nocifensive behaviors, compared to nonconditioned controls, which correlated with reduced population calcium activity of MOR+ vlPAG neurons. Finally, data using a novel enkephalin biosensor to demonstrate the temporal release dynamics of endogenous opioid peptides during acute and chronic pain and placebo analgesia will be shown. Thus, the EAC model combined with cell-type specific viral tools for genetic access to opioidergic neural circuits serves as a strong platform to investigate the malleable nature of pain perception in preclinical pain models and further highlights the vlPAG as a nexus for nociception and endogenous anti-nociception under different motivational need-states.

**Speaker Two**

**Cholecystokinin Facilitates Nocebo Pain Behavior in Mice**

Loren Martin

University of Toronto

Nocebo hyperalgesia, or the experience of enhanced pain due to the expectation of pain or injury, can arise from environmental cues such as surroundings or behaviors of other individuals. Cues from the environment can signal danger or indicate the potential for pain. Observing others in pain or being informed that treatment will cause pain increases pain ratings in human participants. Similarly, when rodents interact with a cagemate in pain, it amplifies pain-related behavior. Further, environmental conditions that signal a threat to safety, such as the presence of an aggressor, can trigger cholecystokinin (CCK) release from neurons. Using social and contextual cues, the Martin lab has recently interrogated the role of CCK, a neuropeptide that underlies nocebo hyperalgesia in humans. Proglumide, a CCK receptor antagonist, blocks socially enhanced pain and decreases neural activation in the periaqueductal gray (PAG) while observing another mouse in pain. Applying proglumide to the lateral PAG (lPAG) prevents socially enhanced pain, while microinfusion of CCK to this region facilitates pain. Silencing ACC neurons and long-range ACC-lPAG CCK projections using chemogenetic and optogenetic approaches blocked socially enhanced pain. Data will also be presented showing that CCK mediates contextually driven nocebo hyperalgesia. These data indicate that CCK receptors in the lPAG, which receive CCK inputs from the ACC, are involved in the descending facilitation of the nocebo response in mice. These findings build a more neuroanatomically precise understanding of the neural mechanisms underlying the social-communicative and environmental enhancement of nocebo pain responses.

**Speaker Three**

**Nocebo: Impact, Mechanisms, and Future Perspectives**

Luana Colloca

University of Maryland

Adverse nocebo responses have emerged as a significant concern in clinical practice and clinical trials, posing potential harm to patients and complicating treatment adherence and efficacy. These nocebo responses manifest as negative outcomes in response to active medical treatments, defying explanations based on the treatment’s pharmacological effects. Although placebo responses are well-documented, the lesser-known phenomenon of nocebo responses can profoundly influence patient outcomes. Nocebo effects can be triggered by various factors, including verbal suggestions, past negative experiences, witnessing others’ negative outcomes, and the broader contextual and environmental factors surrounding a treatment. Recent advancements in medical research have contributed to our understanding of the neurobiological mechanisms underpinning nocebo effects, shedding light on this complex phenomenon. This talk aims to provide a comprehensive review of the studies that delve into different aspects of nocebo effects and responses, exploring their origins and manifestations. Additionally, we will address the clinical implications of nocebo responses, their ethical considerations, and the future directions for research in this field. By elucidating the mechanisms and consequences of nocebo responses, we aim to raise awareness among healthcare practitioners and clinical trial researchers, ultimately improving patient care and the integrity of medical research.

**Where Do We Go From Kere? Exploring Pain and Mental Health for Youth With Rheumatic Diseases**

**Session Chair:** Laurie Proulx

University of Ottawa

**Session Abstract:** Our symposia includes various perspectives including the lived experiences of youth living with chronic pain (Naomi Abrahams) and patient organization leaders (Natasha Trehan), trainee perspectives (Mahta Rafieinia) and researchers (Karine Toupin-April). The panel will be moderated by Isabel Dukes, a young person who lives with chronic pain and arthritis. Naomi Abrahams will share their lived experience living with chronic pain and mental health drawing on her experience in the field of social work. and work as a patient partner in research. Natasha Trehan, founder and executive director of Take a Pain Check Foundation, a youth-led and managed organization that supports youth and young adults with rheumatic diseases. She will share the work of her organization and the Canadian Arthritis Patient Alliance from the Make Rheum for Youth project, including key findings relating to mental health and education and support offered to address pain and mental health. Dr. Karine Toupin April will present her research in developing a patient decision aid (JIA Option Map) to support shared decision making in clinical practice as it relates to pain management and mental health. Our symposia will include a question and answer period and incorporate Slido polling to allow for audience participation. If time permits, we will hold group discussions at tables set up in the symposia room to identify future directions for this topic.

**At the end of this session, participants will be able to**:

- Describe patient decision aids (PDAs) such as the JIA Option Map and describe the evidenced-based information to include in PDAs.
- Identify the lived experience of pain of youth and young people living with rheumatic disease including mental health implications and how patient organizations can help address these needs and translate research evidence.
- Demonstrate how research can help address the needs of youth and young people living with chronic pain and effectively engage patients throughout the research and clinical decision-making process (e.g. patient decision aids).

**Speaker One**

**Making Rheum for Youth With Chronic Pain**

Natasha Trehan with Naomi Abrahams

Take a Pain Check Foundation, University of Ottawa

In 2022, Take a Pain Check Foundation and the Canadian Arthritis Patient Alliance launched a project called “Make Rheum for Youth” in order to better understand the needs of youth and young adults with arthritis. This project is important as youth perspectives are underrepresented in policy and research in Canada, and they experience unique challenges as they navigate life and the transition from pediatric to adult health care. We will present the lived experiences of living with chronic pain (Naomi Abrahams) and explore these experiences drawing on social worker perspective. We will identify mental health challenges identified by the over 65 youth/young adults with rheumatic diseases and identify actions taken through the Make Rheum for Youth project to better support youth and young adults with arthritis in Canada and globally. The survey findings will be presented along with key recommendations and actions we’re taking in this collaboration including as Community Fellows with the Patient and Public Engagement Collaborative at McMaster University.

**Speaker Two**

**Understanding Patient Decision Aids in Support of Evidence-Based Practice**

Mahta Rafieinia

University of Ottawa

The presentation will describe the need for shared decision making and for patient decision aids in pediatric chronic pain. It will also describe the potential of such tools to improve outcomes in pediatric chronic pain, as well as the process used to develop these tools by assessing families’ needs and gathering evidence-based information to inform decision-making. We will give a concrete example with the JIA Option Map, a web-based interactive patient decision aid for young people with arthritis who wish to manage their pain and other symptoms and participate fully in their daily activities. This work has been conducted by an interprofessional research team which includes patient partners, clinicians, and researchers.

**Speaker Three**

**Co-Developing Patient Decision Aids to Address Pain and Mental Health for Youth with Arthritis**

Karine Toupin April

University of Ottawa

The presentation will describe the need for shared decision making and for patient decision aids in pediatric chronic pain. It will also describe the potential of such tools to improve outcomes in pediatric chronic pain, as well as the process used to develop these tools by assessing families’ needs and gathering evidence-based information to inform decision-making. We will give a concrete example with the JIA Option Map, a web-based interactive patient decision aid for young people with arthritis who wish to manage their pain and other symptoms and participate fully in their daily activities. This work has been conducted by an interprofessional research team which includes patient partners, clinicians and researchers.

**From Randomized Trials to Real world: How to Mine the Riches of Healthcare Databases for Better Pain Research and Surveillance**

**Session Chair:** Hermine Lore Nguena Nguefack

Université du Québec en Abitibi-Témiscamingue (UQAT)

**Session Abstract:** Although randomized clinical trials (RCTs) are the gold standard for assessing the benefits and risks of treatments, RCTs do not always reflect real-world clinical practice. For instance, some evidence suggests that nearly two thirds of community individuals living with chronic diseases would not have met eligibility criteria for large RCTs designed to evaluate the efficacy of their treatments. Therefore, longitudinal population-based pain studies that reflect the real world are a crucial complement to RCTs. Canada, due to its universal healthcare coverage, has comprehensive and top-tier administrative healthcare databases on a global scale. Administrative healthcare databases are thus the preferred data source for studying real-world healthcare resource and drug utilization. Although not intended for research, administrative data, such as hospital discharge records and physician billing claims, offer numerous advantages, such as data on entire populations over extended periods with no recall bias. The secondary analysis of such data is often less expensive than primary data collection. Although these data are complex to analyse and present various challenges, including selection biases, researchers have developed different strategies to address these issues (e.g., case-finding algorithms development, linkage with self-reported data). Administrative healthcare databases still remain underutilized in pain research. This symposium will serve as a platform for discussing the added value of administrative healthcare databases for pain research and surveillance. It will also address challenges related to using these data, discuss recent advancements to overcome them, and provide innovative examples of how such data has been used to better understand chronic pain treatment.

**At the end of this session, participants will be able to**:

- Summarize the merits and translational challenges of contemporary pain clinical trials.
- Describe the added value of administrative healthcare databases for real-world evidence and initiatives to improve their access and use by researchers.
- Identify various avenues to maximize the potential of administrative healthcare databases for pain research and provide examples of how such data can be used to better understand intra- and inter-individual variability in chronic pain treatment.

**Speaker One**

**From Randomized Trials to Real World**

Ian Gilroy

Queen’s University

An evidence-based approach is critical for safe and effective pain assessment and management. The introduction of new pain treatments into clinical practice is typically first guided by placebo-controlled, carefully supervised clinical trials conducted under specific conditions (e.g., phase III confirmatory trials). The purpose of such trials is to confirm a given level of efficacy, tolerability and safety thought to be acceptable for use in routine practice. In light of various emerging threats and challenges to the translation of clinical trial results into routine practice, efforts have been focused on reevaluating trial design features (e.g., by the Cochrane Collaboration, IMMPACT and ACTTION) in the interests of improving validity and generalizability of future trials. Aside from obvious generalizability issues with RCTs (e.g., exclusion of patients with psychiatric and other comorbidity, substance use disorders, cognitive problems, washout periods and recruitment from the general population), an important gulf is the contrast between the resource-intensive approach used in RCTs e.g., for analgesic drug dose titration and monitoring (one research nurse per patient) versus relatively unsupervised prescribing approaches in primary care that are obviously limited for practical reasons. Dr. Ian Gilron will review principles and practice of pain clinical trials to highlight merits, and translational challenges, of contemporary trial designs. In particular, necessity and methodology for heavily supervised individual drug dose titration will be reviewed and barriers to translation of these methods into real-world practice will be discussed. Challenges to evaluating the combination of pharmacological and nonpharmacological treatments in clinical trial settings will also be addressed.

**Speaker Two**

**Canadian Administrative Healthcare Databases for Research and Surveillance**

Lisa Lix

University of Manitoba

National initiatives, such as the Public Health Agency of Canada’s Canadian Chronic Disease Surveillance System (CCDSS) and Health Data Research Network (HDRN) Canada, aim to improve the chronic disease research and surveillance environment using administrative healthcare databases. While linked healthcare databases are a valuable resource for population-based observational studies, specialized methodology is needed to extract information from these data and assess its fitness for the intended use. Algorithms (i.e., case definitions) to identify health conditions, such as chronic pain or other measures of interest, such as comorbid health conditions, must be either developed or selected from existing research. These algorithms are typically constructed from standardized codes for diagnoses, procedures, or treatment (e.g., prescription drug treatment) found in administrative healthcare databases. Validation studies are essential to assess the accuracy of algorithms against a reference standard database. Distributed analyses, in which administrative healthcare data are analyzed in each province or territory and the results are combined across these jurisdictions, are used to assess the feasibility of using these algorithms nationally. Dr. Lisa Lix will highlight work across Canada to: (1) assess the accuracy of algorithms for administrative healthcare data, (2) implement these algorithms in multiple provinces and territories, and (3) make algorithms available for use in multi-jurisdictional research and surveillance. New opportunities for using machine-learning methods and multiple sources of administrative healthcare data, including electronic medical records, will be highlighted.

**Speaker Three**

**Expanding the Possibilities of Administrative Healthcare Databases for Chronic Pain Treatment Research**

Anaïs Lacasse

Université du Québec en Abitibi-Témiscamingue (UQAT)

Using administrative healthcare databases for research on chronic pain and its treatment presents several challenges because these data are not intended for research purposes. Firstly, in terms of researchers’ ability to correctly identify individuals living with chronic pain within these data. In fact, the presence of chronic pain is not always reflected by diagnostic codes recorded in the fee-for-service billing system. A second major challenge of using solely administrative healthcare databases is that they do not contain information on the severity of diseases, functional impact, private healthcare utilization, and several important sociodemographic characteristics. Dr. Anaïs Lacasse will discuss recent advancements in terms of the validity of case-finding algorithms, including the added value of using analgesic and coanalgesic prescription claims to refine those algorithms. She will present different chronic pain research infrastructures that allow us to leverage the strengths and overcome the biases of administrative healthcare databases to study the real-world treatment of chronic pain from a biopsychosocial perspective (including the COPE Cohort, the TorSaDE Cohort and the Quebec Pain Registry-PAIR, all of which link administrative healthcare databases to information self-reported by persons living with chronic pain). Their strengths and weaknesses, as well as the feasibility of implementing such infrastructures, will be discussed, along with examples of recent studies that illustrate how such data have been used to better understand intra- and inter-individual variability in chronic pain treatment.

**Democratizing Participant Engagement of Marginalized Communities in Chronic Pain Research: Crafting a Way Forward**

**Session Chair:** Abhimanyu Sud

University of Toronto

**Session Abstract:** The Partnership for the Engagement of Patients in Pain Research (PEPR) is a national partnership that aims to build upon the patient engagement (PE) movement by offering an EDI-D lens (equity, diversity, inclusivity and decolonization). The value of meaningful collaboration with people with lived experiences (PWLE) of inequities is being recognized by health services researchers and funding bodies. Yet, there is no universally accepted framework for PE, and collaboration to date has often not included structurally marginalized populations, despite the prevalence of chronic disease in these communities. This symposium aims to identify how working with PWLE can better reflect the needs and knowledges of those living with chronic pain who have been marginalized by structural oppression and shift our focus from individuals to communities. The multidisciplinary speakers of this symposium are PWLE, senior, midcareer, and trainee researchers and are all members of PEPR. They will highlight how the quality of research on chronic pain can be enhanced through a focus on health equity in policy and care. We will discuss how a shift in conceptualization and practice yields the potential to reframe how certain conditions/diseases are normatively understood, thereby leading to the democratization of research and health for the benefit of people living with chronic pain and marginalization. Our symposium will offer participants an opportunity to build their knowledge and critical thinking through a focus on various aspects of this topic: EDI as applied to PE; a historical and sociological overview of PE; the need to revise existing methodologies; and expanding notions of “who counts.”

**At the end of this session, participants will be able to**:

- Build knowledge and critical thinking around how social science can inform efforts to build equity, diversity, and inclusion as they pertain to PE in chronic pain research.
- Recognize the importance of meaningfully engaging with communities who represent those who are members of historically marginalized groups.
- Open the conversation around how audience members can begin to enact EDI principles in relation to their own research.

**Speaker One**

**Social Science and Community Engagement Approaches to PE**

Fiona Webster

Western University

To date, most approaches to patient engagement in research have focused on the recruitment of individuals, which detracts attention from structural inequities that underpin people’s experiences of being marginalized. In this session, we will explore some of the main tenets drawn from sociology that underpin our approach to community engagement. Specifically, our Partnership involves a series of linked study designs embedded in and guided by key concepts within institutional ethnography (IE), with integration of relevant social theories, such as stigma, marginalization, social exclusion, power, and advocacy. IE refers to an approach to inquiry that uses people’s everyday experiences, or standpoint, as the starting point for an exploration of the often-invisible social relations that underpin or organize their experiences. We combine this with the principles of community engagement using participatory action research (PAR), while also recognizing the limits of PAR. Scholars have noted the compatibility between IE and PAR methodologies in approaching collaboration in ways that reflect the concerns and experiences of people with lived experience. Both IE and PAR inform how we think about and approach the idea of the social organization of health research and people’s involvement in it. Grounded in the experience of participants, IE allows for the creation of research that emphasizes participant-centered principles. This sociological approach aims to produce knowledge about social organization and relations from the standpoint of people and communities occupying places of marginality.

**Speaker Two**

**What is “the Patient Perspective” in Patient Engagement Programs? Implicit Logics and Parallels to Feminist Theories**

Paula Rowland

Wilson Center for Medical Education

Public and patient involvement (PPI) in health care may refer to many different processes, ranging from participating in decision making about one’s own care to participating in health services research, health policy development, or organizational reforms. Across these many forms of public and patient involvement, the conceptual and theoretical underpinnings remain poorly articulated. Instead, most public and patient involvement programs rely on policy initiatives as their conceptual frameworks. This lack of conceptual clarity participates in dilemmas of program design, implementation, and evaluation. This study contributes to the development of theoretical understandings of public and patient involvement. In particular, we focus on the deployment of patient engagement programs within health service organizations. To develop a deeper understanding of the conceptual underpinnings of these programs, we examined the concept of “the patient perspective” as used by patient engagement practitioners and participants. Specifically, we focused on the way this phrase was used in the singular: “the” patient perspective or “the” patient voice. From qualitative analysis of interviews with 20 patient advisers and 6 staff members within a large urban health network in Canada, we argue that “the patient perspective” is referred to as a particular kind of situated knowledge, specifically an embodied knowledge of vulnerability. We draw parallels between this logic of patient perspective and the logic of early feminist theory, including the concepts of standpoint theory and strong objectivity. We suggest that champions of patient engagement may learn much from the way feminist theorists have constructed their arguments and addressed critique.

**Speaker Three**

**An Interpretive Synthesis of PE Frameworks**

Leigha Comer

Western University

In this presentation, we share the findings of an interpretive review (Dixon-Woods 2006) our team, including PWLE, conducted to identify what types of frameworks, guidelines, tools, and models exist to guide and support patient engagement. We first scoped the literature using standard methods, and we were guided by a librarian who helped develop the search terms and identify relevant databases. In total, 73 articles were initially identified. Four investigators abstracted data into a spreadsheet and analyzed 18 to 19 documents each before meeting to discuss. An additional 21 documents were excluded as they did not meet our inclusion criteria, reducing the final number of documents included in this review to 52. Our process of data analysis was approached as an interpretive exercise in which the aim was not the aggregation of data, but rather the production of a critical analysis. To this end, through detailed inspection of the documents, we discussed recurring themes, questions that required further exploration or clarification, significant findings, and critiques. Continual dialogue allowed for the coproduction of a critical analysis through which analytical questions were identified through the initial data extraction process. As a critical approach, data analysis centered around questioning the ways in which the literature constructed and justified patient engagement, conceptualizations of the notion of the patient, the assumptions upon which patient engagement and related frameworks are founded, and the wider history of patient engagement as a particular practice.

**Concurrent Session Two**

Bringing Optogenetics to Pain Therapy – Translational Challenges

**Session Chair:** Yves De Koninck

CERVO Brain Research Center, Université Laval

**Session Abstract:** Optogenetics, the branch of biotechnology which combines genetic engineering with optics to control nerve cells is revolutionizing Brain Science. Therapeutic applications of optogenetic techniques are increasingly plausible. The use of optogenetic tools for human gene therapies represents a completely new paradigm in medicine and is associated with a combination of challenges. For chronic pain, optogenetics can revolutionize neuromodulation approaches by bringing much more specificity in the stimulation target and the ability to provide feedback-control. Yet, translating optogenetics into therapeutics for human applications presents major challenges, with significant barriers. In this symposium we will cover a range of these applications. These include the design of tools that are nonimmunogenic, highly sensitive, and can be activated across tissue, while causing minimal photodamage. Photonics hardware technologies need to combine multiple features, from miniaturization, robustness, and precision to biocompatibility, autonomy, and longevity. Challenges also emerge in implementing gene therapy strategies compatible with targeting the brain, brain regions and cell types. These are not trivial issue when dealing with large human brains. Finally, the prospect of gene-based therapy, brain-machine interface, as well as the potential for such therapy to transform one’s brain, and one’s self, raises an array of ethical, societal, and regulatory challenges that urgently need to be addressed for any translational effort to be successful.

**At the end of this session, participants will be able to**:

- Understand the basics of optogenetics and its potential application to pain therapy.
- Understand the challenges on the technological front for translation to pain therapy.
- Understand the ethical, societal, and regulatory challenges that need to be addressed for the translational effort to be successful.

**Speaker One**

**Technical Challenges for Porting Optogenetics Technologies to Therapy**

Yves De Koninck

CERVO Brain Research Center, Université Laval

Translating optogenetics into therapeutics for human applications presents major challenges, with significant barriers, including: the design of tools that are nonimmunogenic, highly sensitive, and activatable across tissue, while causing minimal photodamage. To enable sophisticated levels of neuronal control, the combined use of optogenetic actuators and sensors must enable closed-loop all-optical neuromodulation, coupled with incorporating decision making computational algorithms. Such closed-loop design must be implemented at multiple levels, i.e. both embedded into the sensors and actuators themselves, but also in the light delivery/collection and control system. Photonics hardware technologies need to combine multiple features, from miniaturization, robustness, and precision to bio-compatibility, autonomy, and longevity.

**Speaker Two**

**Challenges in Viral Based Gene Transfer Therapy for Pain**

Marie-Eve Paquet

CERVO Brain Research Center, Université Laval

Challenges emerge in implementing gene therapy strategies compatible with targeting the brain, brain regions and cell types. Autoregulatory expression mechanisms need to be incorporated into the tools. Although viral vectors such as adeno-associated viruses (AAVs) are showing great potential in human trials, barriers to their general use remain, including immune responses, delivery, and clearance.

**Speaker Three**

**Ethical, Legal and Psychological Boundaries of Optogenetics Therapy**

Jennifer Chandler

Faculty of Law and Brain & Mind Institute, University of Ottawa

Barriers to efficient translation of technologies include restrictive multi-party intellectual property (IP) agreements resulting from the long chains of technical improvements. Each contributor may impose IP conditions that, collectively, preempt future developments. Finally, acceptability and ethical challenges associated with gene therapy and brain-machine interfaces require in-depth dialogs between a broad base of stakeholders, across cultural perspectives.

**The Role of Cannabinoids in Current Pain Practice & Future Implications**

**Session Chair:** Praveen Ganty

University of Toronto

**Session Abstract:** The role of cannabinoids in current pain practice and future implications is a topic of increasing interest and research. The use of cannabinoids has increased exponentially especially since the legalization of cannabis in Canada. Cannabinoids have shown to be effective in various types of pain, including chronic pain, neuropathic pain, and cancer-related pain. Recent data have demonstrated that cannabinoid-based medicines provide a “moderate benefit” in the management of chronic pain. Recently, guidelines for clinical practice guidelines for cannabinoid-based medicines have been formulated and published. These guidelines highlight the potential benefits and appropriate use of cannabinoids in pain management.

It is important for the Pain clinician to comprehend the regulatory environment around the prescription and use of cannabis, and it will be discussed in this symposium by one of the speakers.

However, clinicians who prescribe cannabinoids are aware that the current cannabinoid receptor agonists have displayed only limited analgesic effects, comparable to those of codeine, a weak opioid. One of the reasons could be due to genetic polymorphism, and other drug-drug interactions, limiting the efficacy of cannabinoids. There is ongoing research into the endogenous cannabinoid receptors and ligands that may define a future role for cannabinoids in chronic pain management. We will also touch upon the genetics behind cannabinoid metabolism and drug interactions, in relation to ethnicity.

**At the end of this session, participants will be able to**:

- Comprehend the current prescription and use of Cannabinoids in the Canadian context and the role of genetics in pharmacokinetics of cannabinoid metabolism.
- Explore the guidelines in the prescription of cannabinoids in chronic pain and cancer pain.
- Learn about policymaking and the role of regulatory bodies in the use of medical cannabinoids.

**Speaker One**

**Genetic Insights Into Cannabinoids: Exploring The Human Connection, the Sherlock Holmes WWay**

Praveen Ganty

University of Toronto

Cannabis, a complex plant with diverse pharmacological effects, has gained significant attention for its therapeutic and recreational use. However, the way individuals respond to cannabis can vary widely, and genetics play a crucial role in these differences.

The metabolism of cannabis primarily occurs in the liver, where enzymes, primarily Cytochrome P450 (CYP) family members, break down its psychoactive compound, delta-9-tetrahydrocannabinol (THC). Variations in genes encoding these enzymes can significantly impact the rate at which THC is metabolized. Individuals with specific CYP2C9 and CYP3A4 gene variants may process THC more slowly, leading to prolonged and intensified effects, while others with rapid metabolizing alleles may experience a shorter, milder high. Genetic factors also influence the production of endocannabinoids, endogenously produced cannabinoids, and can influence how these endocannabinoids interact with the endocannabinoid system. Variations in genes such as CNR1 and FAAH can affect endocannabinoid levels and, consequently, an individual’s susceptibility to conditions such as anxiety, pain, or mood disorders.

Understanding these genetic factors can have important implications for personalized medicine, enabling healthcare professionals to tailor cannabis-based treatments to an individual’s unique genetic profile. Furthermore, it can inform responsible use, helping individuals make informed decisions regarding cannabis consumption based on their genetic predispositions. Overall, genetics plays a pivotal role in how cannabis interacts with our bodies, emphasizing the need for further research and individualized approaches in this rapidly evolving field. Ethnic and racial factors may play a role in these interactions. There will also be a short description of the current prescription of cannabinoids across Canada.

**Speaker Two**

**Guidelines on the Prescription of Cannabis in Chronic pain**

Jason Busse

McMaster University

This presentation will review a Health Canada-funded guideline on cannabis for medical purposes and chronic pain. The scope of the guideline was set in a national stakeholder meeting involving patients, clinicians, regulators, and policy makers. Each area of interest was informed by a systematic review of the literature. The guideline was developed from a patient-centered perspective, and patients’ values and preferences were informed through a systematic review and a qualitative study of 52 people living with chronic pain. A 24-member guideline panel comprised of 6 patient partners, 3 methodologists, and 15 clinical experts met to review the evidence and formulate recommendations in seven areas. No panel member had any financial or intellectual conflicts of interest, and the GRADE approach was used to develop all recommendations. We will discuss the resulting recommendations and contextualize them with other recent guidance for cannabis and chronic pain.

**Speaker Three**

**Cannabis and Regulatory Realities: Navigating the Legal Landscape**

Susan Law

University of Toronto

Canada’s approach to cannabis regulation has been a groundbreaking journey, making it one of the world’s pioneers in legalizing recreational cannabis at the federal level.

Canada was the first country among the G7 nations to legalize the recreational use of cannabis. The regulatory framework, known as the Cannabis Act, carefully balances public health and safety concerns with personal freedoms. It establishes strict guidelines for the production, distribution, and sale of cannabis, emphasizing quality control, safety standards, and harm reduction, and provincial regulation and decentralization will be discussed. This speaker will discuss Canada’s regulatory framework that is underpinned by a strong commitment to public education and harm reduction.

Challenges with the Cannabis Act will also be discussed, and collaborative work with other countries such as Australia will also be discussed. Focus will also be on stakeholder perspectives on Canada’s current regulatory framework for medical cannabis and challenges in practice.

**Innovative Methods for Studying Pain Across the Research Continuum: Advances and Implications for Practice**

**Session Chair:** Victoria Abraira

Rutgers University

**Session Abstract:** Advancements in research methods are essential to developing the understanding, assessment, and management of both longstanding and novel issues in pain. Innovations in research methods across the basic, clinical, and implementation sciences not only comprehensively advance the science of pain but also lead to improved, and more equitable access to, pain care for Canadians. This symposium, led by a multidisciplinary (i.e., biology, physiotherapy, psychology) panel of speakers will highlight three innovative research methods across the research continuum. From the basic science perspective, Dr. Victoria Abraira will discuss her use of machine learning and computational models in pain research conducted in mouse models to advance the understanding of pain mechanisms in humans. From the clinical research perspective, Dr. Timothy Wideman will share how the use of a theoretical model of pain can leverage patient feedback to enhance pain assessment, highlighting advancements in how patient perspectives can be integrated to enhance clinical research methods and practices. From an implementation science lens, Nicole MacKenzie will share how a choice-based experimental method can be utilized to understand facilitators of partnership in knowledge mobilization activities within pediatric pain to improve evidence dissemination and implementation practices. This session will feature discussion from moderator Jennifer Daly-Cyr, a patient partner who will emphasize the potential impact these methods have in advancing the discourse of how pain is recognized, conceptualized, and managed. Each speaker will highlight how these innovations along the research continuum can impact practice in pain care.

**At the end of this session, participants will be able to**:

- Describe novel methodological approaches, or novel application of methods, to understanding pain, its treatment, and how to effectively engage partner to mobilize evidence.
- Identify opportunities to potentially apply these methods in other research contexts.
- Consider the critical perspective of people living with pain to interpreting and advancing how these methods are utilized and the questions they may be used to answer.

**Speaker One**

**Utilizing Advanced Techniques and Machine Learning to Decode the Behavioral and Physiological Responses to Pain in Mice**

Victoria Abraira

Rutgers University

Ongoing pain is driven by the activation and modulation of pain-sensing neurons, affecting physiology, motor function, and motivation to engage in certain behaviors. The complexity of the pain state has evaded a comprehensive definition, especially in nonverbal animals. Here, in mice, we used site-specific electrophysiology to define key time points corresponding to peripheral sensitivity in acute paw inflammation and chronic knee pain models. Using supervised and unsupervised machine learning tools, we uncovered sensory-evoked coping postures unique to each model. Through 3D pose analytics, we identified movement sequences that robustly represent different pain states and found that commonly used analgesics do not return an animal’s behavior to a pre-injury state. Instead, these analgesics induce a novel set of spontaneous behaviors that are maintained even after resolution of evoked pain behaviors. Together, these findings reveal previously unidentified neuroethological signatures of pain and analgesia at heightened pain states and during recovery.

**Speaker Two**

**Keeping the “I” in Pain: Novel Conceptual and Methodological Approaches to Access Pain Subjectivity**

Timothy Wideman

McGill University

Pain is defined as a fundamentally subjective experience. And, yet the bulk of the research methods in the field of pain are designed to objectify this experience. We do this by emphasizing the value of quantitative measures of pain and by investing in the potential discovery of pain biomarkers. While quantitative approaches to the study of pain are an essential part of advancing patient care, narrowly focusing on these approaches raises the risk that we fail to understand and address idiosyncratic experiences of pain that are essential to the nature of pain itself. This presentation will present a new conceptual model – the Multi-modal Assessment of Pain (MAP) model – that is specifically designed to facilitate the integration of diverse methodologies to better address the subjectivity of pain. The MAP model provides a framework for synthesizing both qualitative and quantitative data related to pain and aims to advance research on our understanding and management of pain. Recent participatory research on pain-related suffering will be used to illustrate the value of this conceptual model. This work shows how novel qualitative approaches, such as phenomenology, have helped shed new light on previously unaddressed aspects of pain-related suffering. This work also highlights the unique added value of using participatory approaches (which purposefully integrate people living with pain into the planning and implementation of research) to better understand and address pain subjectivity. This presentation will also explore future directions for how qualitative and quantitative approaches can be strategically combined to further advance pain research and practice.

**Speaker Three**

**Asking ‘what’ to Understand ‘how’: Using a Choice Experiment to Determine Factors that Support Partnership in Knowledge Mobilization for Pediatric Pain**

Nicole MacKenzie

Dalhousie University

Engaging in partnership is a best practice in knowledge mobilization activities (KM, i.e., strategies to spread and apply evidence in clinical practice and policy). Inclusion of a variety of partners (e.g., those with expertise in, or make decisions based on, evidence) in KM activities increases their effectiveness and impact. There is limited research, however, on how to engage partners most effectively in KM activities. This is especially the case in the context of pediatric pain, an area with unique considerations for how and where knowledge is shared. Even less is understood about the perspectives of different partner groups (e.g., health professionals, researchers, patients/caregivers) when it comes to engaging in KM processes, potentially hindering the effectiveness of partner engagement in KM. Choice experiments are a rigorous experimental paradigm for examining the preferences and priorities of individuals or groups by studying their decision-making patterns. This presentation will describe a novel application of best-worst scaling, a choice experiment methodology, to study partnership in KM. Specifically, it will demonstrate how a best-worst scaling experiment was used to answer the question of whether different types of partners have difference perspectives on how best to support their engagement in KM activities in pediatric pain. This presentation will highlight key priorities among partner groups when it comes to supporting their engagement in KM activities within pediatric pain and will discuss how these findings can be utilized to improve the development and successful continuity of partnerships in KM activities.

**Concurrent Session Three**

**Taming the Untamable: New Insights Into Mitigating Side Effects of Opioid Use**

**Session Chair:** Tuan Trang

University of Calgary, Hotchkiss Brain Institute

**Session Abstract:** Opioids are a gold standard treatment for acute pain. Despite their utility in treating pain, they possess a myriad of harmful side effects that both limits their effectiveness and can have grave consequences for the patient. Side effects include tolerance, opioid-induced hyperalgesia, and dependence. Although there is an urgent need for novel pain therapeutics, opioids remain the most potent pain medications used in a variety of pain conditions. Mitigating opioid side effects is therefore an important aspect of improving opioid safety and pain treatment outcomes. This symposium brings together clinicians and basic science researchers to highlight clinical interventions and novel research aimed at limiting or treating side effects of opioid use.

**At the end of this session, participants will be able to**:

- Describe major side effects of long-term opioid use.
- Define novel targets being investigated to mitigate opioid-induced hyperalgesia and opioid withdrawal.
- State novel clinical perspectives aimed to limit side effects of opioid use.

**Speaker One**

**Setting the Stage: Can we Reduce the Harms Related to Opioid Use?**

Hance Clarke

Toronto General Hospital, University of Toronto

In the heart of the opioid crisis the pendulum swung very hard in the direction of opioid restriction. The pendulum has begun to be pushed back by patients that are upset about being branded as having an opioid use disorder and harm reductionists insisting that safe supply will keep Canadians alive. This talk will examine opioid prescription practices, trends with respect to the over- and underprescription of opioids, a look into current de-prescribing practices, as well as strategies being employed to help high dose opioid patients transition to opioid agonist therapy. In the final minutes, a few multidisciplinary approaches to supporting patients on opioid therapy and reducing the harms associated with OUD will be presented.

**Speaker Two**

**Targeting the Gastrointestinal Environment as a Treatment for Opioid-Induced Hyperalgesia**

Anna Taylor

University of Alberta

Adverse side effects, including tolerance, hyperalgesia, and the development of an opioid use disorder, are serious clinical challenges for chronic pain management and opioid deprescribing. Opioid receptors are expressed throughout the gastrointestinal (GI) tract, and opioid use both influences gut motility and alters the gut microbiome. Here, we sought to determine if and how the gastrointestinal environment contributes to unwanted symptoms of chronic opioid use. Male and female C57Bl6J mice were treated with escalating doses of morphine (10–40 mg/kg, i.p.) for 4 days after which drug treatment was stopped and mice entered a state of spontaneous withdrawal. We found that opioid induced hyperalgesia persisted for at least 1 week following drug treatment. This time course correlated with an altered gut microbiota composition measured with 16s gene amplicon sequencing and cellular markers of central inflammation. Although we found that germ-free mice (lacking a microbiota) do develop opioid induced hyperalgesia, fecal microbiota transfer from opioid-naïve mice to opioid dependent mice was sufficient to alleviate opioid induced hyperalgesia and neuroinflammation. Increasing GI motility in opioid naïve mice was sufficient to produce opioid induced hyperalgesia, and normalizing GI motility in opioid withdrawn mice attenuated opioid induced hyperalgesia. These results indicate that strategies that target the gut microbiome or GI motility may improve clinical outcomes in prescribing and deprescribing opioids.

**Speaker Three**

**Blocking Microglial Pannexin-1 in the Locus Coeruleus Alleviates Opioid Withdrawal**

Erika Harding

University of Calgary, Hotchkiss Brain Institute

Long-term usage of opioids leads to dependence and persistent use, which is reinforced by significant withdrawal symptoms upon cessation. For many people that use opioids, concern over opioid withdrawal is a major hurdle to tapering or eliminating usage. Previous studies have implicated hyperactive autonomic output as one of the drivers of opioid withdrawal symptoms. The Locus Coeruleus (LC) is a key component of the autonomic nervous system, responsible for releasing most of the central nervous system’s noradrenaline. Here, we sought to understand whether and how the LC may be driving hyperactivity during opioid withdrawal, and whether reducing this hyperactivity can reduce withdrawal symptoms. In both female and male rodent models of opioid withdrawal, we found that spinally projecting LC neurons are hyperexcitable, and that cerebral spinal fluid levels of noradrenaline were significantly elevated. Surprisingly, we found that this hyperexcitability was dependent on activation of microglia, and on Pannexin-1 channels present on these microglia. Pharmacological inhibition or genetic knockdown of microglial Pannexin-1 channels reduced LC neuron hyperactivity and noradrenaline levels in cerebral spinal fluid, and additionally decreased physical symptoms of withdrawal. Together, these findings first highlight how microglial Pannexin-1 channels shift the LC into a hyperactive state during opioid withdrawal, and second provide a therapeutic means through which to decrease this hyperactivity to reduce symptoms of opioid withdrawal.

**What Role for Psychology in Pain Science and Clinical Care? Patient and Scientist Perspectives**

**Session Chair:** Etienne Vachon-Presseau

McGill University

**Session Abstract:** In this symposium, we explore the contrasting perspectives held by scientists and patients concerning the influence of psychological factors on chronic pain. The scientific realm acknowledges the significant impact of psychosocial determinants on the perception and management of chronic pain. Factors such as mood can intensify pain perception, whereas resilience and coping mechanisms can alleviate it. Additionally, an individual’s beliefs and attitudes toward pain can shape their behavioral responses, adherence to treatments, and overall well-being. However, many patients with chronic pain predominantly align with a “body-centric” explanation for their pain. As such, the idea that pain perceptions can be influenced by psychological and societal components is often met with skepticism from the broader public, stemming from an implicit notion that pain might be perceived as a mental construct rather than the result of a distinct pathological process. In this symposium, we will first evaluate the predictive power of psychosocial versus biological factors in the onset of chronic pain using comprehensive biobank data. Subsequently, we will contextualize these findings in relation to public acceptance, culminating with insights from an individual’s lived experience of chronic pain.

**At the end of this session, participants will be able to**:

- Appreciate the roles of psychological factors to predict chronic pain.
- Understand how psychological factors for chronic pain are viewed differently by the public.
- Learn about the perspective of a participant with lived experience.

**Speaker One**

**Predicting Chronic Pain Using Either Psychosocial or Biological Variables**

Etienne Vachon-Presseau

McGill University

Chronic pain is a complex condition influenced by a combination of biological, psychological, and social factors. Using data from the UK Biobank (n = 493,211), I will show that pain is better predicted from psychosocial factors then from biological factors. Using a broad range of psychosocial variables, our data-driven models identified a risk score that classified various chronic pain conditions (area under the curve (AUC) 0.70–0.88) and pain-related medical conditions (AUC 0.67–0.86). In longitudinal analyses, the risk score predicted the development of widespread chronic pain, the spreading of chronic pain across body sites and high-impact pain about 9 years later (AUC 0.68–0.78). Key risk factors included sleeplessness, feeling “fed-up,” tiredness, stressful life events and a body mass index >30. A simplified version of this score, named the risk of pain spreading, obtained similar predictive performance based on six simple questions with binarized answers. The risk of pain spreading was then validated in the Northern Finland Birth Cohort (*n* = 5,525) and the PREVENT-AD cohort (*n* = 178), obtaining comparable predictive performance. These findings show that chronic pain conditions can be predicted from a common set of psychosocial factors, which can aid in tailoring research protocols and improving pain management.

**Speaker Two**

**Is Pain “all in Your Mind”? Examining the General Public’s Views of Pain**

Tim Salomons

Queens University

Clinical psychologists working with individuals in pain often face initial reluctance to engage. In some instances, this is because psychological approaches are perceived as acknowledgment that no cure will be found, in others it might be because psychological referrals are perceived as an indication that the clinical team views pain as psychological in origin. In this talk, I will take a historical perspective on the evolution of our thinking about the psychology of pain, from unfalsifiable “psychogenic” conceptualizations to more rigorous, mechanism-based frameworks. I will also review research from our lab examining how members of the public and individuals living with chronic pain think about pain as a product of the body vs. the mind.

**Speaker Three**

**Psychological Approaches for Pain; a Perspective from a Person Living with Chronic Pain**

Linda Hunter

Person with Lived Experience

Patients living with chronic pain know that pain is far more than a simple sensory experience. It profoundly challenges our sense of self and can alter our emotional and social life. Even after physical reasons are found and corrected for pain management, patients are often resistant to further psychological conceptualizations of their pain, due to a history of such conceptualizations being used to question the validity or even existence of pain. In this talk, I will outline my own journey to find identity as a person living with pain and identify which psychological approaches have been helpful in allowing me to regain power and control and which have been less useful in helping me.

**Mindfulness Programs for Pain: The Evidence Base, and the Move Toward Inclusion, Customization and Equity to Cater to Diversity in Canada**

**Session Chair:** Jacqueline Gardner-Nix

University of Toronto

**Session Abstract:** Evidence is presented to support offering mindfulness-based interventions to clinical populations living with chronic pain, including findings that it could be a cost-saving measure for health budgets. Group mindfulness-based interventions are both self-management and a form of group psychotherapy when offered to those who are referred by physicians and nurse practitioners, and the pandemic has accelerated the feasibility of programs being offered online.

But historically facilitators and participants have not been very diverse, and work needs to be done to promote equity, diversity and inclusion, as well as customizing programs to specific conditions and populations. In mindfulness programs to date, worldwide, most have enrolled 70% to 80% women, reflecting some reluctance by those identifying as male to enter such programs. This is being particularly explored in Veteran programs which involve mostly those identifying as male.

Guidelines have been published to put in place parameters for customized programs to be called “mindfulness based.”

Offering facilitator training to those living with specific pain conditions such as migraine disorder, and from specific populations such as veterans or indigenous peoples, provides the opportunity and necessity of those with lived experience to cofacilitate with regulated healthcare professionals. Training facilitators who are professionals from diverse spaces as well as those with lived experience could attract enrollment by those who are better served by facilitators with whom they can identify. This symposium looks at evidence-base, and the initial experiences of customizing mindfulness programs toward specific conditions and populations.

**At the end of this session, participants will be able to**:

- Describe the role that mindfulness-based interventions can play in chronic pain management and some of the evidence to support them.
- Recognize the importance of adapting mindfulness programs with the input from those living with specific pain conditions or within specific communities.
- Consider the inherent mindful nature of indigenous approaches to pain management, how these connect with western science, and how they can support holistic approaches to healing.

**Speaker One**

**Where and How Does Mindfulness Fit in Chronic Pain Management? The Evidence**

Allen Steverman

Hospitalier de l’Université de Montréal (CHUM)

Mindfulness-based interventions have increasingly been recognized as a valuable therapeutic approach in the management of chronic pain and are included in various guidelines, including diagnosis-specific guidelines (chronic low back pain), and general chronic pain guidelines. We describe how the principles of mindfulness and meditation can support and improve the quality of life for people living with chronic pain. Evidence for efficacy includes the contemplative neurosciences’ investigation of brain activation in meditators correlated with pain tolerance changes, and clinical benefits of group mindfulness-based interventions in populations living with chronic pain.

The principles and application of using a trauma-informed trauma-sensitive lens will be introduced, increasingly important in those living with pain and for those migrating from parts of the world where they experienced trauma. We discuss the relationship between mindfulness and changing lifestyle habits (including diet, physical activity, sleep, and social interactions), and enhancing insight into factors impacting pain, while increasing motivation to adapt, add useful health care interventions, increase personal resilience, and improve outcomes. For those unable to access multidisciplinary clinics these programs, which are now available synchronously online, can motivate participants to be open to multiple strategies and find and engage with them within their localities.

Incorporating mindfulness interventions into clinical management in Canada requires exploration of adapting programs to the needs of many different cultures, communities, minorities, and pain conditions. Feedback from members of those groups will increasingly be sought to develop such programs.

**Speaker Two**

**Co-Facilitating and Co-Adapting in Mindfulness programs as a Lived-Experience Facilitator**

Natanya Mandel

University of Toronto

Two customizations will be discussed in this presentation.

As a person with migraine disorder, I took the training to facilitate mindfulness programs with intent to co-facilitate them for those living with migraine, posttraumatic headache, and headache. Two such online programs have been run in Ontario, and I provide my experience of being a lived-experience cofacilitator working with a health care professional who also lived with migraine disorder.

Enrollment for this program was through Facebook posts, Migraine Canada’s website and bulletins, for which I volunteer. The first program enrolled 18 participants, 15 identifying as female, and was full one month before program start. Most participants had done a mindfulness program before. Customizations and language changes as well as evaluations and outcomes from the program will be discussed, including migraine flare frequency in co-facilitators. Participants were followed for 4 months after the program end and were offered a maintenance program.

Another customization involved lived-experience veterans trained in mindfulness facilitation and cofacilitating in mindfulness groups for veterans and first responders living with chronic pain. Chronic pain is two to three times more prevalent, and also more severe, in veterans than in the general population, and associated with numerous physical, psychological, and social consequences that adversely impact their quality of life. Three such programs were run online during 2020 to 2023, the majority of the participants identifying as male. A brief recorded interview will be shown where veterans report their experience of the intervention, with suggestions for further modifications to be incorporated.

**Speaker Three**

**Mindfulness and Indigenous Populations**

Michael Yellow Bird

University of Manitoba

Indigenous approaches to pain management are often inherently mindful, and co-facilitating Mindfulness programs for such populations with an Indigenous mindfulness facilitator, or having programs fully led by Indigenous facilitators, is essential. The use of the community, special traditional societies, music, healers, and ceremonies, all of which have very strong contemplative and mindfulness aspects, are strategies used in pain management and healing by Indigenous peoples. We can now connect these with western science (neurobiology, genetics, and epigenetics) and know that evidence-base supports contemplative practices influencing changes in the brain, neurochemistry, and the body.

As Mindfulness permeates Western medicine, we can better collaborate on the importance of holistic approaches to heal, and progressively narrow the gap between indigenous and nonindigenous strategies for health care, exchanging knowledge with each other.

But we need to increase the number, visibility, and customizations of mindfulness programs across our land. This should impact our health budgets favorably and, most importantly, will need a collaborative effort by all peoples, while learning from each other.

**Patient Voices in Chronic Pain Management: Harnessing Qualitative Data for Better Care**

**Session Chair:** Deborah Fels

Inclusion and Media Design Center, TRSM, Toronto Metropolitan University

**Session Abstract:** Gaps in the health system make it difficult for patients with chronic pain (CP) to engage with this system and/or communicate their needs. Healthcare decisions and policymakers have indicated that it is critical to include CP patient’s perspectives in their care and allow timely identification of areas where the system is not meeting their needs. Acknowledging the central role of patient voices in influencing pain management decisions and educating various stakeholders on the importance of patient-generated data can increase the quality of care, patient outcomes, and satisfaction. We have developed a digital technology, MyPainMyRecord (MPMR), that enables CP patients to create and share their stories and feedback about their pain care with healthcare providers and others. These data can then be qualitatively and quantitatively analyzed, filtered, visualized and securely shared with their healthcare providers. Perspectives of 10 healthcare providers and patients in our interprofessional multidisciplinary chronic pain management program about MPMR were also collected. Our group from St. Joseph’s Care Group in Thunder Bay and Toronto Metropolitan University, comprising an academician, a CP physiatrist, a pain trainee, and a patient advocate who co-designed the MPMR prototype, will present a summary of the project and initial findings. This presentation will include samples of speech and expressions analytics data, accompanied by a brief demonstration of MPMR. This study is part of a larger project. Each speaker will give a 15-minute presentation, including a patient case study, followed by a 20-minute panel debate on opportunities and challenges of including patient-generated data and narratives in pain management decision-making.

**At the end of this session, participants will be able to**:

- Gain an understanding of the crucial role and usefulness of patient voices and patient-generated qualitative data in chronic pain management decision making.
- Gain knowledge in advocating for a patient-centric approach in chronic pain management by demonstrating how patient narratives and concerns can inform and enhance clinical practice, particularly in a multidisciplinary pain management team.
- Gain an understanding of various cultural perspectives on chronic pain care, as well as how to use technology for practical implementation. This involves recognizing the advantages of combining patient voices and quantitative data, such as increased patient satisfaction, fewer missed visits, and improved interprofessional collaboration.

**Speaker One**

**Introduction to MPMR Project and Patients Living with Chronic Needs and Expectations**

Deborah Fels

Inclusion and Media Design Center, TRSM, Toronto Metropolitan University

MyPainMyRecord (MPMR) is a derivative application that began as a sign language tool for creating accessible websites using linked digital video content. It has been transformed to be an inclusive system where users can record short videos, annotate them, and have them automatically analyzed using artificial intelligence. These analyses can be visualized using various techniques such as word-clouds and text summaries. The video data and visualizations are owned and controlled by the people who create them, but they can also be shared with others such as healthcare professionals (HCP) and family members as desired. Using an inclusive design strategy, MPMR has been designed with a variety of stakeholders and target users in the management of chronic pain fields including people living with chronic pain, and HCP such as physicians, nurses, physiotherapists, social workers, occupational therapists, and personal support workers. Needs analyses and exploratory use methods have informed the development of MPMR from these different perspectives. In our presentation, we will introduce the audience to the MPMR functions and features and work through how the system was designed with HCP and people living with chronic pain.

**Speaker Two**

**Integrating and Assessing Qualitative Nnarratives into MyPainMyRecord (MPMR) for Pain Care Decision-making**

Hadi Shojaei

Northern Ontario School of Medicine U, and St Joseph Care Group

Population-based surveys demonstrate that over 1 in 5 persons suffer from chronic pain, ranking among Canada’s top three reasons for seeking medical care. Several barriers hinder improving chronic pain (CP) management and outcomes, including collecting long-term data, assessing intervention responses, and addressing communication challenges between patients and healthcare providers (HCPs). Ensuring continuity of care for CP patients has become increasingly important, especially during long intervals between in-person appointments. On many occasions, when we see patients in a follow up visit, patients cannot recall or explain their associated symptoms, or reduction/improvement in affected physical, mental, and/or daily living functionality. Our innovative technology, MPMR, is designed for recording and sharing healthcare related information, observation and qualitative narratives from CP patients. HCP attitudes regarding the utility of the MPMR platform design for allowing CP patients receiving pain care services will be discussed. Once a patient records their audio/video during their symptoms flare up, HCP can not only access them promptly to address the issues, but also integrate and incorporate appropriate interventions within the HCP-patient team. Some of the examples of assessing qualitative narratives which have important diagnostic and therapeutic roles include sleep issues, bowel and bladder problems, moods, energy level and functions, stress and anger, libido, medication’s effects, weight changes, other sensory abnormalities like hearing, vision, etc. Having all this data analyzed and available to the CP management interprofessional team, will allow them to create a comprehensive treatment plan that is adaptive to the patient’s needs at the time of visit.

**Speaker Three**

**Giving Voice to Chronic Pain Patients: Engagement of CP Patients Living in Remote Communities in Their Health Care Decision Making**

S. Fatima Lakha and Jeff Milner

Inclusion and Media Design Center, TRSM, Toronto Metropolitan University

Millions of Canadians experience chronic pain. Communication gaps within healthcare systems make it difficult for CP patients to express their needs and concerns. Currently, tracking patients’ chronic pain journey occurs when an HCP is present to hear, integrate, and record that input. Patient-reported outcomes incorporated into healthcare records are becoming recognized as important for guiding care management and adaptation. In our research, we engaged in a user-centered design approach and conducted semi-structured interviews with 10 patients with chronic pain (CP) who were receiving treatment at an interdisciplinary pain clinic. Before the interviews, we collected information about participants’ demographics, familiarity with technology, and willingness to share data. Our primary objective was to explore the integration of qualitative data and narratives authored by CP patients with digital health data using our innovative MPMR technology platform. In partnership with St. Joseph Care Group (SJCG) in Thunder Bay, a prominent provider of pain care services, we aimed to demonstrate how this platform could enhance case management in rural communities, with a particular focus on understanding and incorporating the patient perspective. We will summarize our current research findings, including concerns, questions, and negative and positive experiences.

Additionally, a patient advocate will share his experience with MPMR ‘s development to provide valuable insight. Furthermore, we will provide an update on the current status of the prototype and outline our future directions. This will prompt a panel discussion on integrating patient-generated data and narratives into pain management decisions, with a focus on opportunities and challenges.

**Concurrent Session Four**

**Hot Topics Trainee Presentations**

**At the end of this session, participants will be able to**:

- Describe the latest research in pain mechanisms and clinical care; and

- Critique and evaluate emerging topics in pain research.

**Poster Presentation: Toward a Computational Understanding of Chronic Pain**

M. Lacombe-Thibault

Université Laval, Center Interdisciplinaire de Recherche en Réadaptation et Integration Social (CIRRIS)

**Introduction**: According to a learning account of pain, pain should be modulated to optimize learning. The aim of this study was to test this hypothesis by assessing the influence of the informational value of pain on the subjective perception of pain in healthy participants. We expected that the participants’ pain perception would be greater when pain was surprising and the environment uncertain.

**Methods**: Fifty healthy participants (28 F, 22 M; mean age of 24,38 years old) took part in an experimental task in which they had to learn to predict the associations between high or low tones and high or low painful electric stimuli. At each trial, a tone was presented and was followed by a painful stimulus. Participants had to indicate as quickly as possible if the pain was high or low. The probabilities related to the tone-pain association changed throughout the task and remained stable for several trials or changed quickly to create periods of low and high environmental uncertainty.

**Results**: Model-free analyses confirmed that participants took longer to answer when pain was unexpected and that expectations had a significant effect on subjective pain perception. Model-based analyses using a hierarchical Bayesian learning model also suggested that trial wise computational estimates of expectations and uncertainty estimates were significantly related to pain perception.

**Discussion/Conclusions**: This research reinforces the established link between pain and learning, demonstrating that pain is adaptively adjusted to enhance learning efficiency. These findings offer a basis for exploring how learning about pain could be altered in the context of pathological pain conditions.

**Poster Presentation: Understanding the Impact of Demographic and Psychological Factors on Pain Variability in Adults with Chronic Pain: The Circa Pain Project**

E. Lamoureux

Department of Psychology, Université de Montréal, Canada

**Introduction**: Chronic pain is a complex experience marked by substantial inter- and intra-individual pain variability. While chronic pain is commonly associated with symptoms of psychological distress including anxiety and depression, little research has examined the impact of demographic and psychological factors on pain fluctuations among this population.

**Methods**: Canadian adults living with chronic pain (*n* = 555, age = 57.0 ± 13.0 years, 84.5% woman) were recruited via partner advocacy organizations, social media, and clinic outreach to participate in the Circa Pain Project. Participants completed a baseline questionnaire measuring pain type and duration, pain intensity, anxiety, and depression, and an electronic diary tracking their pain intensity 3 times per day for 7 days. Multiple linear regression models were used to test whether age, gender, anxiety, depression, and pain catastrophizing significantly predicted pain variability (*SD* of pain scores) among participants who had completed at least 80% of the diary entries.

**Results**: Among the examined variables, anxiety was the only significant variable negatively associated with pain variability (B = −0.03, *p* = .01). Sensitivity analyses showed that results remained unchanged if only those with 50% of diary completion were considered.

**Discussion/Conclusions**: **The** current findings provide evidence that individuals who report fewer anxiety symptoms experience greater pain variability (for example, pain that follows a circadian rhythm) than individuals whose pain remains more constant over time. This is in line with previous reports showing that those with a circadian pain rhythm report less depression and opioid consumption than those who report stable pain intensity. Further research is needed to examine the temporality of this relationship.

**Poster Presentation: Expression and Localization of 5-HT Receptors in the Dorsal horn of Rat and Human Spinal Cords**

C. Murray-Lawson

Carleton University, The Ottawa Hospital Research Institute Department of Neuroscience

**Introduction**: Serotonin, also known as 5-HT, is a promising potential target system for treating chronic pain. Although rodent studies and preclinical investigations on psychedelics, as well as 5-HT receptor agonists and antagonists, suggest a connection between serotonergic signaling and spinal pain processing, the specific mechanisms underlying this relationship remain largely unknown. The superficial laminae of the dorsal horn are pivotal in nociception; however, the distribution of 5-HT receptors in the spinal cord remains unexplored in human samples, with no consideration given to potential sex differences.

**Methods**: This study employs a multidimensional approach to map the distribution of 5-HT receptors in rat and human spinal pain circuits. Through immunohistochemistry, qRT-PCR, and single-cell/nuclei RNA sequencing, we systematically investigated the spinal expression of all 5-HT receptor subtypes as well as localization of 5-HT2C in dorsal horn circuits, with analyses across spinal cord region, sex, and species.

**Results**: Our findings suggest that the 5-HT2C receptor is highly expressed and preferentially localized to the superficial laminae of the dorsal horn in both rat and human spinal cord and other serotonin receptor subtypes are expressed densely within the dorsal horn. Furthermore, we are investigating potential sex differences and variations in receptor expression and localization across thoracic and lumbar spinal cord sections.

**Discussion/Conclusions**: The 5-HT2C receptor, along with other 5-HT receptor subtypes, exhibit a dense expression in the superficial laminae of the dorsal horn in both rat and human spinal cords. This pattern suggests an involvement of spinal serotonin transmission in the modulation of pain.

**Poster Presentation: Chronic Pain, Prescriptions, and Peril: Predicting Risk Trajectories**

H. Lore Nguena Nguefack

Université du Québec en Abitibi-Témiscamingue

**Introduction**: Quantifying medication-related risk is important for enhancing patient safety, informed decision making, and responsible medication use awareness. This study aimed to describe risk trajectories associated with medication use for chronic pain.

**Methods**: A cohort study was conducted using the TorSaDE Cohort, which links 2007–2016 cycles of the Canadian Community Health Survey with Quebec health administrative data; 8,760 adults reporting chronic pain and covered by the public prescription drug insurance two years post-survey completion were selected. The monthly risk associated with medication use was calculated, using the Medication Quantification Scale 4.0, a single score obtained by assigning risk weights to each analgesic/coanalgesic used. Growth Mixture Modeling was applied to identify subgroups (trajectories) of individuals with similar patterns of risk over time.

**Results**: Five risk trajectories were obtained: **1)** low-stable risk over time (*n* = 2,988;34.1%), **2)** moderate-stable risk (*n* = 2,801;32.0%), **3)** moderate risk with a marked increase over time (*n* = 601;6.9%), **4)** high risk with a marked decrease over time (*n* = 597;6.8%), **5)** high-stable risk (*n* = 1,773;20.2%). The “high-stable risk” group was the one with the highest proportion of females (73.0%) and excessive polypharmacy (≥10 medications: 37.9%). The “moderate-stable risk” group showed the highest proportion of ≥65-year-old participants (62.7%) and the “high-risk-decreasing” group showed the highest proportion of participants with some/most activities prevented because of pain (49.6%) and fair/poor perceived general health (56.9%).

**Discussion/Conclusions**: Different subgroups of people experience varied risk patterns associated with their use of pain medications. It is essential to better understand the particularities and determinants of adverse trajectories to effectively prevent them.

**Poster Presentation: Microglial Activation is Regulated by Circadian Rhythms in Chronic Neuropathic Pain**

C. O’Connor

Department of Biomedical and Molecular Sciences, Queen’s University

**Introduction**: Microglia have been shown to be drivers of pain hypersensitivity in the spared nerve injury (SNI) model of neuropathic pain in mice. Following SNI, microglia in the dorsal horn of the spinal cord proliferate and transition from a homeostatic phenotype to a pro-inflammatory phenotype. These cellular changes are accompanied by behavioral changes as mice develop mechanical and thermal (cold) allodynia. Clinical studies have shown that pain can be rhythmic in various chronic pain states. However, it remains unknown whether microglia exhibit circadian rhythmicity in chronic pain states.

**Methods**: To investigate this gap in knowledge, male and female C57BL/6 mice received spared nerve injury, with tissues collected at 3, 7, 10, 14, 28, and 84 days following injury. Animals were sacrificed at ZT2 and ZT14, corresponding to 2 hours after the start of the light or dark phases. The spinal cord was stained for markers of homeostatic and pro-inflammatory microglia, and images were taken to create 3D renderings of the cells.

**Results**: We found changes in both microglial morphology and activation state (using markers Iba1, P2RY12, CD68) in both the naïve and injured state. During peak periods of microglial activation following SNI, which occur between 7 and 14 days following injury, microglia in the dorsal horn took on a more pro-inflammatory phenotype at ZT2, and a more homeostatic phenotype at ZT14.

**Discussion/Conclusions**: Further understanding of microglial activation states across male and female mice, and during the circadian cycle, may provide new insight into mechanisms regulating their activity and function in the pathophysiology of chronic neuropathic pain.

**Poster Presentation: Identifying Subtypes and Pain Trajectories in Chronic Overlapping Pain Conditions**

C. Tanguay-Sabourin

Faculty of Medicine – University of Montreal, AECRP – McGill University, CRIGUM – University of Montreal

Introduction: Chronic pain often coexists with other forms of pain and comorbid conditions, referred to as Chronic Overlapping Pain Conditions (COPCs). These conditions are challenging to study due to their diverse etiologies and clinical presentations. This study aimed to identify homogeneous subtypes of COPCs and understand their distinct pain spread trajectories using unsupervised machine learning.

**Methods**: We analyzed data from 81,600 individuals with chronic pain who completed the UK Biobank online pain questionnaire including pain ratings across 12 distinct body sites. To characterize pain spread, we used the unsupervised machine learning algorithm merging disease progression with clustering to obtain probabilistic spatiotemporal partitioning. Subtypes were then compared based on their pain spread trajectories, diagnoses, and nonpain symptomatology.

**Results**: We identified four different subtypes (S1-4) associated with distinct putative trajectories of pain spread. These trajectories were characterized based on the spread (Number of Pain Sites, *R^2^ *= 37–38%) and intensity (Worst Pain Rating; *R^2^ *= 32–40%) of pain but could also capture the impact of pain (Brief Pain Interference; *R^2^ *= 25–34%). These subtypes exhibited varying predominant body sites, combinations of pain diagnoses (nociceptive, neuropathic, and nociplastic), and multisystem symptomatology (e.g., cardiological, respiratory, GI among others). Importantly, across all subtypes, the trajectories of pain spread were associated with greater signs of neuropathic symptoms localized at their most bothersome pain site (DN4; *r* = 0.23–0.40).

**Discussion/Conclusion**: In conclusion, our data-driven model shows distinct patterns of pain spread and accompanying symptomatology within COPCs. Understanding these trajectories holds the potential to inform the etiology of chronic pain and provide insights into effective pain management strategies.

**Beyond Sensation: Pain Modulation in Cognitive and Motivational Contexts**

**Session Chair:** Mathieu Roy

McGill University

**Session Abstract:** Cognitive and motivational factors play a pivotal role in shaping our pain experience. This symposium will explore three distinct, yet interrelated research topics, unveiling insights from novel experimental methods probing cognitive and motivational modulation of pain. Roy will outline existing pain and cognition models and spotlight literature gaps, setting the stage for panelists’ discussions.

**Value-Based Pain-Cognition Interactions**: Pain-cognition interactions are traditionally thought to be salience-driven. However, these models don’t account for pain’s inherent value. Moayedi will present the results of experiments using noxious and innocuous somatosensory stimuli showing that while innocuous stimuli affect all tasks, pain interference in cognition varies with the task’s value, suggesting a value-based framework for understanding pain-cognition interactions.

**Motivational Influences on Pain Perception**: Coll will discuss recent neuroimaging findings showing how the brain assigns a distinct value to potential future pain, influencing decision-making processes toward stimuli. Moreover, when pain conveys new information about the environment, pain ratings and physiological responses are amplified, highlighting the intrinsic link between pain modulation, learning, and control.

**Harnessing the Experience of Flow and Passion as a Powerful Analgesic**: Distracting tasks may reduce pain, but can flow experience and passion amplify this analgesia? Deldar suggests that engaging in preferred activities, such as gaming or passionate activities such as chess significantly reduces pain ratings, emphasizing the role of individualized flow experiences and passion in pain modulation.

This symposium aims to explain the interplay between pain, cognition, and motivation, offering insights that might open the door for novel interventions targeting the cognitive and motivational aspects of pain.

**At the end of this session, participants will be able to**:

- Gain insights into the nature and mechanisms of value-based task priority in pain and cognition.
- Learn how engaging in enjoyable activities can be a strategic tool to alleviate pain, emphasizing the role of individual flow experiences and passion in pain management.
- Learn about the latest experimental approaches and results in the experimental study of the impact of cognitive and motivational factors on pain perception.

**Speaker One**

**A Value-Based Model of Pain-Cognition Interactions**

Massieh Moayedi

McGill University

Pain can interfere with cognitive processes, resulting in forgetfulness, an inability to focus, and difficulties in abstract thinking, problem solving and decision making. A fundamental gap in our understanding of pain is the mechanism of this interference, which would serve as a therapeutic target for pain. It is unknown whether pain’s inherent salience interrupts ongoing tasks (i.e., distraction), or whether it competes with the ongoing task based on its inherent value, or both. If the former is true, any salient stimulus should exert the same effects as pain on tasks. If the latter is true, the value of pain can determine the extent of its interference – task priority depends on perceived value. We aimed to determine how pain impacts cognition using a value-based framework. Participants performed two cognitive tasks while receiving either a noxious chemical-heat stimulus, or a tonic nonpainful electric stimulus. The tasks had conditions with high-value rewards ($1.00/correct response) and low-value rewards ($0.05/correct response). Salience was matched across stimuli and was confirmed with skin conductance responses. Compared to baseline, noxious heat selectively affected performance based on task value: it slowed reaction times on the low-value (*p* < .05) but not high-value task. The iso-salient electric stimulus affected performance on both tasks, regardless of value (*p* < .05). Although a nonpainful sensation impacted both tasks, the effect of pain depended on the value of each task. This suggests the impact of pain on cognition depends more on its value than its salience.

**Speaker Two**

**Motivational and Informational Influences on Pain Decisions and Perception**

Michel-Pierre Coll

Laval University

Pain not only captures our attention but also prompts us to address potential sources of injuries. Its significance for survival extends beyond immediate reactions, enabling us to anticipate and avoid potential threats by assigning an aversive value to potential pain-inducing cues or actions. In this talk, I will delve into two primary research areas involving healthy human adults: the neural representation of the value of future pain and the modulation of pain perception based on its informational value about the environment. Using neuroimaging and computational modeling, I will demonstrate that the brain represents potential future pain with a distinct neural signature, predicting participants’ decisions to approach or avoid pain for monetary rewards. Furthermore, I will show that subjective pain perception and associated physiological responses are increased when pain conveys novel information. Collectively, these findings highlight the deep connection between pain modulation, learning, and control, and offer insights into how disruptions in these processes might contribute to persistent pain in clinical conditions.

**Speaker Three**

**Can we Boost Analgesia by Harnessing Flow Experience and Passion?**

Zoha Deldar

McGill University

Pain demands our attention as a natural protective response, but distraction can reduce it. Although cognitively demanding tasks can minimize pain, their often unpleasant and effortful nature limits their efficacy and acceptability. However, imagine if diving into an enjoyable task or a passionate hobby could not only entertain us but also alleviate pain. In this talk, we will unpack the interaction between enjoyment, flow, and passion, exploring their capacity to boost analgesia.

We will explore two research areas: in our first study, participants played their preferred and nonpreferred digital games while undergoing thermal pain stimuli, revealing a substantial reduction in pain unpleasantness in the favored game compared to the less preferred (*p* = .02) and typical cognitive tasks (*p* < .001). Meanwhile, our second study with advanced chess players found a significant alleviation in pain intensity and unpleasantness while solving chess puzzles compared to a standard cognitive task. Together, these findings highlight the crucial role inter-individual differences in flow experience, passion, and enjoyment play in pain reduction. Future interventions might strategically utilize these facets, sculpting more effective, cognitively distracting pain interventions.

**Real-World Implementation of Digital Health Solutions Spanning the Continuum of Care to Improve Pain Management in Canada**

**Session Chair:** Lynn Cooper

Person with Lived Experience

**Session Abstract:** We know that waiting for chronic pain care has serious consequences; untreated pain in childhood increases risk for lifetime chronic pain and mental health and substance use issues, and adults with chronic pain report decline in functioning and quality of life while waiting, especially in underserved populations. Funded by Health Canada, the Power Over Pain Portal is a direct response to the Action Plan for Pain in Canada to improve access to timely, equitable, and patient-centered pain care and provide educational supports to people living with pain. In this symposium we will introduce the Youth and Adult *Power Over Pain Portal (PoP)*. The PoP Portal is a virtual portal codesigned by a diverse group of researchers, clinicians, and people living with chronic pain to provide rapid access to evidence-based virtual stepped care interventions for chronic **pain across the lifespan**. In line with the Stepped Care 2.0 framework, the PoP Portal facilitates continuous outcome monitoring to allow adjustments to treatment recommendations and applies advanced data analytical approaches to recognize patterns related to engagement and recovery and develop predictive models to tailor treatment plans. This session will be an interactive opportunity to learn about the stepped care framework and showcase examples of implementation across various contexts. This session is chaired by a person living with pain who is also a cocreator of the Power over Pain Portal. The lived experience will be woven throughout the symposium to ground the real-world relevance for people living with pain accessing pain care.

**At the end of this session, participants will be able to**:

- Learn how to leverage a stepped care 2.0 framework to ensure timely access to pain resources across the continuum of care such that people have access to the right care at the right time.
- Learn how digital health solutions are used by youth with chronic pain in primary and tertiary care settings.
- Explore implementation strategies and outcomes (health and health system outcomes), as well as patient impact around implementation of the Power Over Pain Portal.

**Speaker One**

**Development and Implementation of a Stepped Care Framework for the Organization and Delivery of Evidence-Informed Programming for the Management of Pain, Mental Health and Substance Use Concerns**

Josh Rash

Memorial University of Newfoundland

Stepped Care 2.0 offers a framework for integrating resources across a continuum of care in a manner that is graded in intensity. Integrated resources can range from educational, self-directed, peer-support, individual or group programming, to specialist interdisciplinary care. The system is resiliency-based, grounded in recovery-oriented principles, and self-corrective. Care is driven by need, preference, and readiness of the service-user to engage. Care can be stepped up or down in intensity, based on results from continuous outcome monitoring. This presentation will focus on how the core components of an integrated stepped care model for pain management can be implemented that leverages mental health and substance use resources. Efforts underway in the province of NL to implement a provincial stepped care framework for pain management will be used as a case example with particular attention to the role that Power Over Pain plays in augmenting pain self-management resources. I will conclude by drawing alignment between the stepped care framework and two goals outlined in the Action Plan for Pain in Canada: 1) improving access to timely, equitable, and person-centered care; and 2) ensuring equitable approaches for populations disproportionately impacted by pain.

**Speaker Two**

**Co-Design, Development, and Implementation of the Power over Pain Portal for Youth with Chronic Pain**

Jennifer Stinson

The Hospital for Sick Children, Toronto

Pediatric chronic pain is a rising public health concern. Given its high prevalence, there will never be capacity to treat everyone within tertiary care clinics and early tailored intervention could prevent lengthy wait lists and functional deterioration. The PoP Portal will empower youth from across Canada with rapid access to early, flexible, and individualized chronic pain interventions to improve quality of life and functioning. The PoP Portal is a comprehensive virtual platform codesigned during the pandemic by healthcare providers and a diverse group of Canadian youth living with chronic pain. It includes (a) participant registration, (b) self-assessment tools (completed bi-weekly to help guide the participants’ choice of interventions), and (c) evidence-based virtual educational (pain neuroscience; Step 1), web- and app-based Cognitive Behavioral Interventions for pain management (Step 2) and virtual peer support/navigation (Step 3) that are delivered as per the Stepped Care 2.0 model (i.e., based on participant needs, readiness, and preference). In this presentation, I will: (a) describe the creation and co-design of the Youth PoP and highlight the involvement of people with lived experience; and (b) share current implementation and evaluation efforts of the PoP Portal in primary care and those waiting on tertiary care pain clinic wait-lists. PoP is a one stop shop that will improve rapid access to evidence-based chronic pain care and enhance clinical outcomes for youth with chronic pain.

**Speaker Three**

**Going Beyond Pilot Studies: Implementing Evidence-Informed Pain Solutions for Real-world Impact**

Rachael Bosma

University of Toronto

One in five Canadians suffer from chronic pain, and while evidenced and effective nonpharmacological interventions exist to support them, access to effective pain interventions remains a major barrier in Canada. Recognizing the lack of resources to meet the needs of people with chronic pain and the potential of digital health interventions to address issues around access to appropriate care, we created the Power Over Pain Portal, an online platform integrating evidence-based solutions to support people with chronic pain. In 8 months over 12,000 people became active users on the PoP Portal. Many users (15%) access not only pain resources, but also mental health and substance use health resources. To date, 85% of patients on the waitlist of pain clinics approached about the PoP Portal willingly attended an orientation session, 100% of people who attend an orientation session continued to use the portal 4 weeks later, 100% would recommend the use of the portal to others, and 80% planned to continue to use the portal beyond the 4-week follow up.

This presentation will describe how we have moved beyond the creation of an innovative solution, to understand how PoP can be effectively integrated into care settings. We will discuss implementation strategies and highlight how the use of the Power Over Pain Portal has decreased wait times, resulted in deferred appointments, enhanced access to care in clinical settings and in rural and remote areas, and led to healthcare savings.

**Beyond Checking the Box: Practical Lessons for Integrating EDI into your Pain Research, Teaching, and Service**

**Session Chair:** Rebecca Pillai Riddell

York University

**Session Abstract:** Integrating justice, equity, inclusion, and diversity into one’s practice as a pain researcher is an ongoing learning journey. This workshop invites pain researchers to examine their practices as clinicians, as teachers, and as academic citizens. Each speaker will share their expertise with the goal of having participants take away practical knowledge to improve how they approach their work. Dr. Anna Hood (Psychology, University of Manchester) will present on an innovative theoretical model of how racism-based traumatic stress impacts the initialization and perpetuation of chronic pain using research from sickle cell populations. Dr. Jaris Swidrovich (Pharmacy, University of Toronto) will critically evaluate the concept of Evidence-Based Medicine from a decolonial and Indigenous lens. He will challenge tenets of dominant systems of knowledge generation and transmission to facilitate a greater appreciation of Indigenous approaches to knowledge relating to medicine. Dr Rebecca Pillai Riddell (Psychology, York University) will present on a new open-access, bilingual EDI learning platform called POLARIS—a **P**lace of **O**nline **L**earning for the **A**djudication of **R**esearchers **I**nclusively and **S**upportively. Funded by the Canada Research Chair’s EDI stipend and York University, POLARIS sets out to provide practical skills training for researchers to adjudicate more inclusively and supportively. Ultimately, this workshop sets out to teach and inspire pain researchers about what they can do as teachers, clinicians, and academics to transform places within the academy and medical settings by re-imagining structures that integrate and celebrate diverse ways of knowledge generation and practice.

**At the end of this session, participants will be able to**:

- Discuss the impact of racism-based traumatic stress on pain inequities through the lens of the RESTORATIVE conceptual model and describe actionable steps to mitigate healthcare barriers in clinical practice.
- Analyze Evidence Based Medicine (EBM) through decolonial and Indigenous lenses, fostering a greater understanding of strategies that honor Indigenous medicines and healing practices.
- Utilize more just practices when serving on peer review committees for hiring or awards because of a more informed understanding of “inclusive excellence.”

**Speaker One**

**How do we Bridge the Equity Gap? Examining Racism as a Driver of Pain Disparities**

Anna Hood

University of Manchester

A wealth of research indicates that people from racialized groups consistently receive less adequate treatment for acute and chronic pain than White people, even after controlling for age, gender, and pain intensity. People who have experienced interpersonal discrimination are at higher risk for exposure to institutional/systemic racism as it is in the “groundwater.” Racism-based traumatic stress (RBTS) conceptualizes the significant emotional and mental injury caused by frequent uncontrollable racist experiences and encounters as stress-inducing with the potential to produce traumatic responses and the added burden of a sociopolitical context where threats are constantly present but generally invalidated by the dominant White society. In this presentation, Dr Hood will examine the potential impact of RBTS through the lens of the Racism Exposure and Trauma Accumulation Perpetuate Pain Inequities – Advocating for Change (RESTORATIVE) model. The RESTORATIVE model integrates the models of racism and pain and demonstrates how the shared contribution of trauma symptoms (e.g., racism-based traumatic stress and PTSD) maintains and perpetuates chronic pain for racialized groups. Utilizing the RESTORATIVE model, we will describe specific, actionable steps for clinicians to use cultural humility and openness and be advocates, facilitators, and leaders whilst mitigating trauma and injustice. We will share knowledge gained from working with young people living with sickle cell disease and discuss how to confer a sense of safety and trust whilst discussing validation and empathy for pain as potential pathways toward restoration and equitable change.

**Speaker Two**

**Laying the Groundwork for Decolonization, Indigenization and Reconciliation in Pain Research and Practice**

Jaris Swidrovich

University of Toronto

The drastic privileging of Western knowledge is problematic in the healthcare context where epistemic racism (domination of knowledge) and systemic racism (when systems treat people differently based on ethnicity or race) work together to delegitimize Indigenous research and evidence, which impacts resource allocation and access to culturally appropriate care for people in pain. A decolonial and Indigenous lens will be applied to Evidence Based Medicine (EBM) along with strategies for health professionals and researchers to honor, celebrate, and integrate Indigenous knowledges and practices into pain research, teaching, and service.

**Speaker Three**

**Who Am I to judge? An Introduction to POLARIS—a Place of Online Learning for the Adjudication of Researchers Inclusively and Supportively**

Rebecca Pillai Riddell

York University

It is clear that simply sitting in a workshop will not lead to large-scale transformative change in academic structures that house pain research and practice. It is the application of that knowledge to concrete action that dismantles and rebuilds systems known to perpetuate bias. POLARIS (the Place for Online Learning for the Adjudication of Researchers Inclusively and Supportively) was funded by the Canada Research Chairs Equity Diversity and Inclusion Fund and York University to create free, bilingual open-access learning modules by professors for professors to support better practices when we judge one another in hiring or award contexts (https://www.yorku.ca/research/project/polaris/). Highlights of key learning from these modules will be presented to provide structure and focus as to how to be a better adjudicator of other researchers. As we welcome more diverse researchers into the academy and uphold the concepts of community-based or patient-oriented research, researchers must understand what inclusive excellence is and how to use appropriate metrics to adjudicate inclusive excellence.

**Concurrent Session Five**

**Sex Differences in the Immune Cell Regulation of Pain Resolution**

**Session Chair:** Bradley Kerr

University of Alberta

**Session Abstract:** There is a growing recognition that immune cells present in, and around injured tissue are not only mediators of pain sensitizing factors but also play an important role in tissue healing, repair, and recovery from pain. More recently however, it has been recognized that important sex differences exist in the complement of immune cells present after injury and in the signaling, they engage in to regulate pain sensitivity. This symposium will bring together three leading laboratories studying the neuroimmunology of pain to discuss recent findings highlighting some of these critical differences in the immune response to injury between males and females and their implications for determining whether pain resolves or becomes chronic. The speakers will highlight a novel, sex-specific role for immune cell production of endogenous opioids and the circadian regulation of heat sensitivity. They will also discuss recent findings regarding sex differences in immune cell driven neural plasticity and how this might impact on the ability to resolve pain appropriately. Finally, we will hear about specific populations of T cells and how these cell types can influence pain resolution in a sex specific manner.

**At the end of this session, participants will be able to**:

- Evaluate the most recent data regarding the immunology of pain and pain resolution.
- Consider how biological sex can influence processes such as tissue healing and pain resolution.
- Describe sex differences in the response of specific classes of immune cells as it relates to pain and plasticity.

**Speaker One**

**Inflammation, Plasticity, and Pain: A Tale of Sex, Death and Potassium**

Bradley Kerr

University of Alberta

The intricate interplay between inflammation, excitability, and plasticity in dorsal root ganglion (DRG) neurons is a relatively unexplored area of research. We have begun to examine how secreted factors from innate immune cells impact on the physical plasticity of primary sensory neurons of the DRG and how this relates to pain. Our findings reveal that an early phase of increased neuronal activity is critical for the engagement of plastic processes, and that neuronal excitability profiles are linked through time to the structural phenotype of individual neurons. Importantly, biological sex appears to be critical for these responses. Our findings suggest that neurons from biological females are inherently more “plastic” when challenged with inflammatory stimuli either in vitro or using in vivo models of inflammatory disease. We have also been able to identify a critical role for voltage gated potassium channels in this process. In this talk we will review the data examining the increased propensity for neural plasticity in the face of inflammatory challenges by females and discuss the relationship this has to increased pain sensitivity in various inflammatory diseases.

**Speaker Two**

**Sexually Dimorphic Circadian Rhythms of Innate IImmune Cells Regulate Nociception Via the Opioid System**

Nader Ghasemlou

Queen’s University

The sensation of painful stimuli are known to follow a 24-hour rhythm in various chronic pain states, including multiple sclerosis, migraine, and diabetic neuropathy. Recent evidence has shown that endogenous circadian rhythms, and not sleep, are responsible for control of baseline thermal nociception in naïve males. However, the mechanisms underlying these changes in sensory function remain unknown. We therefore studied changes in mechanical and thermal (hot/cold) sensitivity in naïve male and female C57BL/6 J mice using an integrated neuro-immune approach. Our findings reveal a sex-specific effect for nociception rhythmicity, controlled specifically by the endogenous opioid system. Using immune cell-specific depletion strategies, we found that rhythms of innate myeloid immune cells are responsible for changes in thermal but not mechanical sensitivity. Ongoing studies in male and female humans and *Drosophila melanogaster* (the common fruit fly) also show a rhythmicity to thermal stimuli in the naïve state. This work highlights the important contribution of neuro-immune interactions, not only in acute and chronic pain states but also in the resting state, and points to potential therapeutic strategies harnessing endogenous circadian rhythms to reduce pain, such as chronotherapy.

**Speaker Three**

**Interleukin-10-Producing Monocytes Contribute to Sex Differences in Pain Resolution in Mice and Humans**

Jaewon Sim

Michigan State University

Pain is closely associated with the immune system, which exhibits sexual dimorphism. For these reasons, neuro-immune interactions are suggested to drive sex differences in pain. However, our understanding of the impact of peripheral neuro-immune interactions on sex differences in pain resolution remains limited. Here, we have shown, in both a mouse model of inflammatory pain and in humans following traumatic pain, that males had higher levels of interleukin (IL)-10 than females, and these higher IL-10 levels were correlated with faster pain resolution. Following injury, we identified monocytes as the primary source of IL-10, with IL-10-producing monocytes being more abundant in males than females. In a mouse model, we demonstrated that neutralizing IL-10 signaling through antibodies, genetically ablating IL-10R1 in sensory neurons, or depleting monocytes with chlodronate all impaired the resolution of pain hypersensitivity in both sexes. Furthermore, manipulating androgen levels in mice reversed the sexual dimorphism of pain resolution and the levels of IL-10-producing monocytes. Our results highlight a novel role for androgen-driven peripheral IL-10-producing monocytes in the sexual dimorphism of pain resolution. These findings add to the growing concept that immune cells play a critical role in resolving pain and preventing its transition into chronic pain.

**The Quebec Back Pain Consortium: Studying Low Back Pain from Multiple Perspectives**

**Session Chair:** Jean-Sébastien Roy

Université Laval

**Session Abstract:** Persistent low back pain (LBP) is the leading cause of years lived with disability worldwide. Current therapeutic interventions are often either not effective or are associated with undesired consequences. These concerns are further amplified by the current opioid epidemic, resulting in an enormous public health crisis. The need for an interdisciplinary team is critical to study the contributing factors of persistent LBP. Experts from diverse disciplines including kinesiology, neurobiology, epigenetics, genetics, psychology, neuropsychology, neurophysiology, rehabilitation, spinal and cerebral imaging, trajectory of care, epidemiology, patient pain experience and healthcare communication gathered and created the Quebec Back Pain Consortium to address this challenge. The main objective of the consortium is to establish a cohort of individuals with acute LBP (aLBP) and follow them for two years to identify biopsychosocial factors predicting who will develop persistent LBP and who will recover. Moreover, our cohort study provided the opportunity to create a large databank of participants interested to participate in additional “satellite” research projects linked to the consortium. Researchers can use the databank to foster their recruitment. The main goal of this symposium is twofold:

- To describe the Quebec Back Pain Consortium prospective cohort study including the multiple outcomes collected, the time-points used, and the sample size recruited;
- To present satellite projects associated with the consortium studying LBP from diverse perspectives.

Specifically, the projects selected will discuss the impact of genetics, low back pain definition and brain organization in LBP trajectory, and the effect of various types of exercise, and noninvasive brain stimulation to reduce pain and disability.

**At the end of this session, participants will be able to**:

- Describe the Quebec Back Pain Consortium prospective cohort study including the multiple outcomes collected, the time-points used and the sample size recruited.
- Present satellite projects associated with the consortium studying LBP from diverse perspectives.
- Discuss the impact of genetics, low back pain definition and brain organization in LBP trajectory, and the effect of various types of exercise, and noninvasive brain stimulation to reduce pain and disability.

**Speaker One**

**Transition of Acute to Chronic Low Back Pain: A Role for Acute Pain Definition and Genetics**

Caroline B. Meloto

McGill University

Accurately predicting the transition of acute LBP (aLBP) to chronic LBP (cLBP) is needed to enable strategies to avoid chronicity and the personal and societal burdens that follow. One of the reasons for this inaccuracy may be the lack of standardization of the aLBP definition across studies. Following up on our group’s previous work showing that adopting different definitions leads to diverging baseline group characteristics, we explored how using different aLBP definitions impacts the profile of risk factors linked to the transition to cLBP. We have found that (1) the ability to predict this transition, as determined by AUC curves, increases as the stringency in the aLBP definition adopted also increases, and (2) the risk factors of this transition change from one definition to another. In this symposium, we aim to make the point that harmonizing the guideline for aLBP definition is required to facilitate the identification of consistent risk factors. Another reason for this inaccuracy arises from the fact that risk factors typically assessed in pain studies (e.g., pain intensity, disability, negative affect) explain only a relatively small proportion of the large interindividual variability in the risk of transition to cLBP. Incorporating genetics in the form of polygenic risk scores into risk prediction models is likely to increase predictive accuracy, as is seen for other common polygenic health conditions. In this symposium, we will present evidence to support the use polygenic risk scores as a means of identifying individuals at higher risk of transitioning to cLBP.

**Speaker Two**

**Unveiling the Neurobiological Mechanisms of Physical Exercise Therapy for Chronic Low Back Pain**

Mathieu Roy

McGill University

Chronic Low Back Pain (CLBP) poses a global health challenge with limited effective treatments. Physical exercise is a promising intervention for CLBP, but its underlying mechanisms remain poorly understood. Previous research has associated CLBP with heightened functional connectivity between the nucleus accumbens (NAc) and medial prefrontal cortex (mPFC), implicating reward circuit dysfunction in the maintenance of pain. Our study aimed to elucidate the cerebral mechanisms underlying physical exercise’s impact on CLBP. We enrolled 57 participants in a 14-week physical exercise program or a wait-list control group. Physical exercise led to significant reductions in pain and disability. Neuroimaging analyses revealed a substantial decrease in NAc-mPFC connectivity, suggesting the disruption of a potential pain-amplifying mechanism. Additionally, intrinsic connectivity within critical pain and emotion processing brain regions, including the superior frontal gyrus and insular cortex, decreased. Concurrently, we observed elevated brain-derived neurotrophic factor (BDNF) levels and altered immune cell gene expression, implicating metabolic function, mitochondrial function, CNS plasticity, immune response, and stress regulation. Importantly, changes in these pathways correlated with pain improvement and NAc-mPFC connectivity alterations. Our comprehensive study underscores physical exercise’s potential in alleviating CLBP. Furthermore, our findings highlight the relevance of reward circuit dysfunction in pain conditions and hint at exercise’s ability to ameliorate associated emotional and stress-related symptoms.

**Speaker Three**

**Noninvasive Brain Stimulation Combined with Motor Control Exercise in Chronic Low Back Pain: The ExTraStim Randomized Controlled Trial**

Hugo Massé-Alarie

Université Laval

**Introduction**: Motor control exercises (MCE) improves pain and disability, but its effect remains modest. Repetitive transcranial magnetic stimulation (rTMS) is a promising technique that alleviates neuropathic pain but its effectiveness to treat CLBP is uncertain. Combining rTMS with MCE may help to address both central and nociceptive factors contributing to CLBP.

**Objective**: To compare the effectiveness of rTMS, sham rTMS, rTMS+MCE and sham rTMS+MCE on pain intensity and disability.

**Methods**: 140 participants with CLBP were randomly allocated into four groups (rTMS+MCE, sham rTMS+MCE, rTMS, sham rTMS) to receive 10 sessions of their assigned intervention within 8 weeks. Pain intensity and disability were measured at baseline, 4 and 8 weeks, at 3 and 6 months. Linear mixed models were computed using fixed factors rTMS, MCE, and Time.

**Results**: No interaction including Time was significant (rTMS × Time, MCE × Time, MCE × rTMS × Time) both for pain and disability. Main effects of Time were significant for pain (*p* < .001) and disability (*p* < .001). Regardless of groups, pain decreased by a maximum of 1.8 pts at 8 weeks (*p* < .001), and disability improved by a maximum of 9% at 4 weeks (*p* < .001).

**Conclusions**: The efficacy of noninvasive brain stimulation to improve pain or disability in CLBP is not supported. Conversely to literature, the addition of exercises over sham or active rTMS did not result in better clinical results. A strong placebo effect due to noninvasive brain stimulation may explain these results. Future studies should determine if different subgroups of participants respond to the different combinations of interventions.

**Implementing Large-Scale System-wide Healthcare Solutions for Chronic Pain Management: Implementation Science, Knowledge Mobilization, and Complexity-Informed Research from Across Canada**

**Session Chair:** Elena Lopatina

Alberta Virtual Chronic Pain Program, Alberta Health Services

**Session Abstract:** In Canada, comprehensive chronic pain care employing biopsychosocial approaches remains scarce and is difficult to access. Among the reasons for this shortfall are the challenges in developing, implementing, and sustaining interventions that would meet the needs of diverse patient groups in diverse settings. These interventions are complex, comprising multiple interconnected components, and often are not limited to one clinic, department, or patient group but are large-scale, designed to facilitate system-wide changes.

As illustrated by implementation science, it is challenging to roll out such interventions. Our session will highlight exemplary initiatives implementing large-scale system-wide healthcare solutions for chronic pain management across Canada, including:

1. The Quebec Digitally-Enabled Integrated Care Program, which embeds innovative technologies throughout the patient care trajectory to enhance access to interdisciplinary care, personalized education, and self-management in Quebec;
2. The Alberta Virtual Chronic Pain Program, which aims to improve chronic pain management at the community and primary care levels of care across Alberta; and
3. The Pediatric Pain Management Health Standard, which aims to improve pediatric pain management in hospitals across Canada.

The session will commence with panelists presenting their respective cases to share insights on the successes, roadblocks, and key takeaways. Following this, the moderator will weave in concepts from implementation science, knowledge mobilization, and complexity-informed research and invite panelists to partake in a lively discussion to contrast and compare the distinct challenges and opportunities that arise when implementing large-scale, system-wide healthcare solutions at the local, provincial, and national levels. The discussion will conclude by contemplating considerations and strategies pivotal for the success of future initiatives.

**At the end of this session, participants will be able to**:

- Identify success factors, challenges, and key takeaways from the exemplary large-scale system-wide healthcare solutions for chronic pain management from across Canada.
- Compare and contrast the unique challenges and opportunities present when implementing large-scale system-wide healthcare solutions for chronic pain management at the local, provincial, and national levels.
- Propose considerations and strategies for future large-scale system-wide healthcare solutions for chronic pain management.

**Speaker One**

**The Quebec Digitally-Enabled Integrated Care Program**

Regina Visca

McGill University

The Quebec Digitally-Enabled Integrated Care Program embeds innovative technologies throughout the patient care trajectory to enhance access to collaborative interdisciplinary care and personalized education and self-management across all levels of care, especially in remote areas of Quebec. Interleaving technology with service delivery enables timely and continuous access to 1) interdisciplinary asynchronous and synchronous primary care consultation; 2) remote patient monitoring including real-time personalized self-management strategies and clinical advice; 3) self-directed self-management; 4) real-time clinical information during different points of care; and 5) coordination of care. The program is being codesigned with providers, decision makers, and patients, using the principles of learning health systems to improve access, integration, continuity, coordination, and communication.

Regina Visca is the network coordinator for the McGill RUISSS Center of Expertise in Chronic Pain, where she co-leads the development and implementation of digitally-enabled care and is a member of the provincial advisory committee to the Ministry of Health responsible for overseeing the development and implementation of the Quebec Chronic Pain Action Plan. She is also a PhD Candidate at the McGill University. Her work focuses on the role of digital health in supporting integrated and person-centered care delivery for patients living with chronic pain, using a complexity-informed lens in applying implementation science and evaluation methods to explore how best to integrate technology. During the symposium, Ms. Visca will reflect on her firsthand experience with the Quebec Digitally-Enabled Integrated Care Program and her extensive expertise in bridging evidence-based innovations, policy advocacy, and learning collaboratives to transform health systems.

**Speaker Two**

**The Alberta Virtual Chronic Pain Program**

Magali Robert

Alberta Virtual Chronic Pain Program, Alberta Health Services, University of Calgary, Calgary Chronic Pain Program

The Alberta Virtual Chronic Pain Program is a province-wide initiative aiming to enhance accessibility of pain education and self-management support at the community and primary care levels of care. This Program is currently being developed and implemented in Alberta. Patients and healthcare providers are actively engaged in the Program’s development. The Program is innovative in many ways. It will offer multiple services and delivery methods, to allow easy, tailored access to care. The Program will enable self-referral and offer self-directed resources and facilitator-led group-based programs as well as peer support. In parallel, support for Primary Care Providers will be offered by facilitating access, providing consistent messaging, and supplementing their care with virtual resources through the Program. The Program places special emphasis on assisting patients in remote and rural Alberta.

Dr. Magali Robert is the Medical Lead of the Alberta Virtual Chronic Pain Program. As part of this role, she is currently engaged in all aspects of the Program’s development and implementation. Dr. Robert is also a Professor in the Cumming School of Medicine, University of Calgary and the Medical Director of the Calgary Chronic Pain Program. During the Symposium, Dr. Robert will discuss the development and implementation of the Alberta Virtual Chronic Pain Program, while also reflecting on her extensive clinical and leadership experience in chronic pain management.

**Speaker Three**

**The Pediatric Pain Management Health Standard**

Katie Birnie

University of Calgary, Solutions for Kids in Pain, Alberta Children’s Hospital

Solutions for Kids in Pain (SKIP) is a national knowledge mobilization network aiming to improve evidence-based management of pain in children through coordination and collaboration. SKIP partnered with the Health Standard Organization (HSO) to develop the world’s first national health standard for pediatric pain management, the Pediatric Pain Management Health Standard. This National Standard of Canada published in April 2023 (CAN/HSO 13200:2022) outlines 34 specific criteria with accompanying guidelines detailing how organizational leaders and health care teams can deliver quality, equitable pain care for children from birth to 19 years less one day in any hospital setting.

In her role as the Associate Scientific Director of SKIP, Dr. Katie Birnie led the development of the Pediatric Pain Management Health Standard and is currently leading the knowledge mobilization work to facilitate the Standards’ adoption across Canada. Dr. Birnie is also a Clinical Psychologist at Alberta Children’s Hospital and Assistant Professor in the Department of Anesthesiology, Perioperative and Pain Medicine at the University of Calgary. In her research, Dr. Birnie creates new knowledge, synthesizes existing evidence, and facilitates knowledge translation to inform health practice and policy to improve the assessment and management of acute, postsurgical, and chronic pain in children and adolescents. As such, Dr. Birnie not only brings to the Symposium her firsthand experience with developing and mobilizing the Pediatric Pain Management Health Standard, but also her valuable research and clinical expertise.

**Peer Support Needs Support: Building Capacity for Chronic Pain Peer Support Across Canada**

**Session Chair:** Yaadwinder Shergill

Power Over Pain Network

**Session Abstract:** Peer support is a process of giving and receiving support founded on shared lived experience and key principles of respect, shared responsibility, and mutual agreement of what is helpful (Mead et al., 2001). Peer support has been described as “disruptive innovation” (Pat Deegan). Substantial research has demonstrated the mental and physical health benefits of peer support. In recent years, peer support (particularly online support) for people with chronic pain has become more widely accessed as part of the stepped care continuum.

This symposium will include discussion, present findings, and bring together multiple perspectives on peer support including:

1. Advocacy and collaboration on shared needs and resources for peer support across Canada
2. Experiences of peer facilitation, use of intentional lived experience and group structure
3. Emerging research from a longitudinal study on the impact of online peer support groups

**At the end of this session, participants will be able to**:

- Identify shared needs and opportunities of peer support organizations across Canada and learn how collaboration among national pain peer led support group organizations enables them to build capacity and better service people living with pain.
- Gain an understanding of the experiences of a Pain BC peer facilitator and the group structure in order to assist others interested in group development.
- Share outcomes of emerging research on the impact of online peer support.

**Speaker One**

**National Action on Peer Support for Chronic Pain**

Virginia McIntyre

People in Pain Network

Pain Canada is a national action network, supported by Pain BC, created in response to Health Canada’s 2021 report An Action Plan for Pain in Canada. One of Pain Canada’s goals is to expand the reach of stepped care and pain self-management by building capacity for pain peer support programs across the country. Over the past year Pain Canada has convened leaders from L’Association Quebecoise de la Douleur Chronic (AQDC), People in Pain Network, Power over Pain Portal, and Corrections Canada to identify shared needs, resources, better understand challenges and opportunities for collaboration, and codevelop materials for peer support facilitators. In this presentation delivered by Virginia McIntyre from People in Pain Network and codeveloped with Melanie McDonald from Pain BC/Pain Canada, we will share the outcomes of this work including:

- Development of evidence-based materials for pain peer support organizations nationally
- Provision of shared training opportunities for peer facilitators’ continuous educational development
- Creation of a peer facilitator’s competency framework that could be used as a guide; and
- Supporting organizations to build quality improvement practices into practice

The opportunities and challenges of this work will be discussed.

**Speaker Two**

**Cracked AND Whole: How Facilitating Peer Support Groups Helped me Put the Pieces Back Together**

Jennifer Lorca

Pain BC

Research demonstrates the significant benefits of peer support for people with chronic pain, including building stronger social connections, improved daily functioning, and better pain self-management. Although there is research on the benefits of peer support, there is little that focuses on the role and experiences of peer facilitators. The purpose of this presentation will be to explore the experience of facilitating a peer support group, intentional use of lived experience, and the structure of peer groups at Pain BC.

Pain BC is a registered charity offering a wide range of psychosocial supports for people who live with pain, including online, peer-delivered support groups. The purpose of these groups is to provide people who live with pain with emotional support, education, and social connection to improve their well-being. The groups’ guiding principles are anti-oppression and trauma-informed, person-centered care, and peer-led. Groups are led by volunteer peer facilitators who intentionally use lived experience to cocreate a space for participants to feel connected to a supportive community. Groups are offered biweekly by geographic region. Recent groups were developed to provide peer support for people with pain who identify as Black, Indigenous and People of Color (BIPOC), young adults and LGBTQ2 + .

In this session, peer facilitator Jennifer Lorca, BSN, will discuss the following:

- Their experience facilitating peer support groups
- The structure of the groups
- The role of role of intentional use of lived experience
- What they are continuing to learn in their role as a facilitator

**Speaker Three**

**Zooming In and Out: Participants of Online Peer Support Groups and Their Outcomes Over Time**

Susan Holtzman

University of British Columbia

Social support is a critical determinant of health among people living with chronic pain (PLCP). Yet, PLCP frequently report high levels of social isolation, loneliness, and a perceived lack of understanding from their social networks. Online peer support groups are an increasingly common intervention used to increase support and wellbeing among PLCP, and can help to overcome some of the barriers associated with in-person participation. Although studies highlight a range of benefits of online peer support, much of the research remains cross-sectional, leaving gaps in our understanding of the long-term benefits of group participation and predictors of patient engagement/disengagement over time. In a collaboration between Pain BC and Dr. Holtzman’s research team, a longitudinal study has been launched to examine the impact and effectiveness of Pain BC’s online peer-delivered support groups. Study participants complete baseline online questionnaires comprised of standardized measures of psychosocial factors (e.g., loneliness, pain self-efficacy) and demographic questions, as well as follow-up surveys at 3, 6, 9, and 12-months. To date, 50 participants have completed baseline questionnaires and 30 have completed the 3-month follow-up. Participants have been predominantly middle-aged women with longstanding chronic pain who are not employed. This presentation will report on the results of the 3- and 6-month follow-up data to provide insights regarding change in function over time. Challenges in participant recruitment and feedback regarding the groups’ barriers and strengths will also be discussed as a means of informing the ongoing sustainability and expansion of this service.

**Concurrent Session Six**

**Genetic and Genomic Approaches can help us Understanding the Underlying Cause of Chronic Pain**

**Session Chair:** Greg Neely

University of Sydney

**Session Abstract:** It is not entirely clear why some people experience chronic pain and others do not, however risk of chronic pain is highly influenced by a patient’s genetic code. New genomic technologies like genome-wide association studies (GWAS), DNA sequencing, and transcriptomic profiling allow researchers to begin to answer this question at the molecular level. This session will highlight recent efforts to identify the genetic basis of pain diseases. Analysis of the DNA protein coding sequence of hundreds of thousands of people highlights SLC13A1, a sodium/sulfate symporter, which may represent a novel pain therapeutic target. RNA sequencing of patients who underwent acute to chronic pain transition reveals a protective role of inflammatory response against this process, suggesting new therapeutic targets involving stimulation of immune response for chronic pain prevention/reversal. Alternatively, by combining human patient sequencing with functional validation in a model organism, we have identified SETDB2 as a novel human gene causing congenital insensitivity to pain. Overall, unbiased human genomic approaches help us uncover why some people are more at risk of developing chronic pain disease, and this knowledge can efficiently guide the development of new classes of chronic pain therapies to manage pain disease.

**At the end of this session, participants will be able to**:

- Recognize the utility of DNA sequencing approaches in large human biobank and their potential to identify new gene candidates contributing to pain states.
- Recognize the role of inflammatory response in acute to chronic pain transition and how this knowledge can guide new therapeutic approaches.
- Recognize the utility of a functional genomic approach to identify and characterize genes that control human pain perception.

**Speaker One**

**Rare Variant Analyses in Large-Scale Cohorts Identified Sodium/Sulfate Symporter SLC13A1 Associated with Chronic Pain**

Luda Diatchenko

McGill University

Here, we performed gene-based rare variant analyses in 200,000 human subjects in the UK biobank whole-exome sequencing database for investigating nine different chronic pain states and validated our findings in three other large-scale databases. Our analyses identified the SLC13A1 gene coding for sodium/sulfate symporter associated with chronic back pain and multisite pain at the genome-wide level and with chronic headache, knee, and neck and shoulder pain at the nominal level. Seven loss-of-function rare variants were identified within the gene locus potentially contributing to the development of chronic pain, with two of them individually associated with back pain and multisite pain. These two rare variants were then tested for replication in three other biobanks, and the strongest evidence was found for rs28364172 as an individual contributor. Transcriptional analyses of Slc13a1 in rodents showed substantial regulation of its expression in the dorsal root ganglia and the sciatic nerve in neuropathic pain assays. Our results stress the importance of the SLC13A1 gene in sulfate homeostasis in the nervous system and its critical role in preventing pain states, thus suggesting new therapeutic approaches for treating chronic pain in a personalized manner, especially in people with mutations in the SLC13A1 gene.

**Speaker Two**

**Stimulation of Inflammatory Response as New Therapeutic Target for Chronic Pain**

Lucas Lima

McGill University

Our recent findings from transcriptomic analysis of low back pain patients, pharmacological manipulation on animal models, and big data analysis from the UK Biobank, reveal evidence that acute inflammatory response has a protective against chronic pain, with this effect being neutrophil-driven. Moreover, our data suggests that conventional anti-inflammatory treatments may promote pain chronification. These results indicate the need to reassess current protocols for managing acute pain with anti-inflammatory methods and at the same time opens new potential pain therapeutic targets that bolster the immune response rather than dampening it. In this session, we will present transcriptomic data from chronic low back pain patients who underwent a therapeutic exercise program. Our results reveal that the pain-relieving benefits of exercise are, at least in part, mediated by its immunomodulatory effects. Additionally, we will also present preclinical data on potential immunostimulant therapies for pain relief (neutrophils, S100 proteins, and activated serum), as well as the use of inflammation itself as a therapeutic tool.

**Speaker Three**

**SETDB2 Mutations are a Novel Conserved Cause of Congenital Insensitivity to Pain**

Greg Neely

University of Sydney

Chronic pain affects hundreds of millions of people world-wide and current therapies do not adequately address pain for most patients. To identify core regulators of pain perception we combined functional screening in fruit flies with human exome sequencing of extreme pain patients. From this, we identified 99 conserved genes that control sensory neuron development or function, including the predicted histone methyltransferase SETDB2, which we confirm as a new cause of congenital insensitivity to pain (CIP). We generated SETDB2 KO mice and found they also exhibit an age-dependent decline in acute pain perception, primarily mechanical nociception. We next generated humanized SETDB2 CIP mutant mice, and these animals also recapitulate the patient CIP phenotype. To identify the molecular cause of CIP in SETDB2 CIP mutant mice, we performed single cell sequencing, and identified changes in ROS and translational stress programs across all sensory neurons, as well as broad transcriptional disarray in low threshold mechanoreceptors. A mechanical-specific phenotype was confirmed in C-fibers by electrophysiology. Mechanistically, we found SETDB2 is not a histone methyltransferase but forms a complex with P53 and DAXX which can drive sensory neuron necroptosis, and dysregulation of this pathway may contribute to the observed pain phenotype. Overall, our conserved functional genomics approach highlights SETDB2 as a critical new pain gene and understanding how SETDB2 controls pain perception can inform how we treat these CIP patients or other individuals at risk of peripheral neuropathy.

**Predicting the Transition to Chronic Pain: Insights from Across Biopsychosocial Systems**

**Session Chair:** David Seminowicz

University of Western Ontario

**Session Abstract:** Chronic pain is a major global health problem with few effective treatments. A key limitation is a lack of clinically meaningful biomarkers that can predict pain outcome, facilitate early intervention and provide new targets for treatment and prevention of chronic pain. Recent research efforts have sought to identify biomarkers across biopsychosocial systems using large-scale longitudinal studies of the transition from acute to chronic pain. This presentation will highlight data from these studies. Using both persistent human pain models and clinical pain populations, this symposium will interweave and integrate findings from separate studies to identify biomarkers common to the transition to chronic pain. Data will span the biopsychosocial spectrum from molecular pathways to cortical activity to the psychosocial. Each presentation will conclude by identifying opportunities for clinical translation and future directions.

**At the end of this session, participants will be able to**:

- Describe predictive markers of chronic pain from across different biopsychosocial systems.
- Identify mechanisms involved in the transition from acute to chronic pain.
- Recognize potential new targets for the prevention of chronic pain.

**Speaker One**

**A Novel Brain Mapping Based Biomarker of Orofacial Pain Severity**

David Seminowicz

University of Western Ontario

**Introduction**: This presentation will highlight data from the PREDICT study, in which the aim was to validate a novel brain-based biomarker of pain severity in a model of temporomandibular disorder (TMD).

**Methods**: 159 healthy individuals were enrolled, and 150 of those participants had complete datasets that were included in the analysis. The model of TMD was induced via injections of nerve growth factor to the masseter. Pain was assessed for the following 30 days via diaries and laboratory assessments. Peak alpha frequency (PAF) assessed with electroencephalography (EEG) and corticomotor excitability (CME) assessed with transcranial magnetic stimulation were acquired repeatedly in the first five days of TMD model onset. We used predictive analyses to determine whether high and low pain sensitive individuals could be classified via brain measures alone or in combination with other variables.

**Results**: We could classify high and low pain sensitive individuals with an accuracy of 89%. The combination of PAF and CME provided the best prediction and the addition of demographic and psychological variables did not improve the model accuracy.

**Discussion**: These findings have direct clinical relevance: since pain at first onset TMD is predictive of the development of chronic TMD, we can potentially use this brain biomarker to identify patients likely to develop chronic TMD and intervene early.

**Speaker Two**

**Brain-Based Biomarkers in the Transition from Acute to Chronic Low Back Pain**

Siobhan Schabrun

University of Western Ontario

**Introduction**: This presentation will highlight data from the Understanding persistent pain where it resides (UPWaRD) prospective longitudinal trial of the transition from acute to chronic low back pain.

**Methods**: The study followed 120 individuals with acute low back pain over 6 months as they either i) recovered from their pain or ii) transitioned to chronic pain. A range of mechanisms including sensorimotor cortex excitability, human assumed central sensitization, and psychosocial factors were assessed at baseline, 3 months, and 6 months.

**Results**: Factors that accurately predicted the transition to chronic pain will be discussed, and this will be followed by the results of a causal inference analysis identifying factors that caused chronic low back pain.

**Discussion**: The presentation will conclude with a discussion of potential therapeutic strategies to target these mechanisms.

**Speaker Three**

**Immune Dysregulation During the Transition from Acute to Chronic Low Back Pain**

Luke Jenkins

University of Technology Sydney

**Introduction**: This presentation will highlight results from a novel multi-omic analysis that aimed to test the hypothesis that dysregulation of the tryptophan/kynurenine metabolism pathway drives transition from acute to chronic low back pain.

**Methods**: This study followed 59 individuals with an acute episode of low back pain from the Understanding persistent pain where it resides (UPWaRD) prospective longitudinal study over 3 months as they either recovered or reported ongoing pain. Serum samples from these participants were assessed for global differences in protein expression as well as targeted assessment of key proteins and metabolites involved in the tryptophan/kynurenine pathway using mass spectrometry.

**Results**: Sex differences were evident in the expression of proteins related to immune control. Differentially expressed proteins that correctly classified 93% of males and 90% of females with ongoing pain at three months will be discussed followed by results of the targeted protein/metabolite analysis.

**Discussion**: The presentation will conclude with discussion of how strategies targeting distinct immune system processes in males and females could interfere with transition from acute to chronic low back pain.

**Solutions for a CRiPpling Syndrome: An Update on New Diagnostic Categories, Identifying Imitators, and Therapeutic Breakthroughs for CRPS**

**Session Chair:** Nimish Mittal

University of Toronto

**Session Abstract:** Complex Regional Pain Syndrome (CRPS) is a debilitating and enigmatic pain disorder that has confounded medical professionals for decades. Diagnostic ambiguity is one of the primary obstacles in managing CRPS. This symposium seeks to explicate recent advances in CRPS diagnostics and therapeutics, in accordance with the 2019 Valencia update, which addressed ambiguities in the Budapest CRPS criteria application in research and clinical settings. The presentation will delve into an often-overlooked aspect of CRPS – numerous conditions that replicate its symptoms – as it is essential for effective management to differentiate CRPS from imitators. Experts will examine the most recent research and strategies for identifying and differentiating these imitators, providing necessary resources to healthcare professionals to avoid misdiagnoses and provide individualized care. Attendees will acquire an understanding of the indications and evidence for novel pharmacological interventions and procedures including neuromodulation, and alternative therapies that have the potential to revolutionize treatment of CRPS management and provide much-needed relief to those suffering from CRPS.

Each of the 3 speakers will deliver a 15–20-minute presentation, followed by a video of an individual with lived experience of CRPS who has consented to tell her story and discuss her treatment. Subsequently, the chair will facilitate a panel debate on the opportunities and obstacles in current CRPS classification framework and therapeutic interventions, followed by audience queries and an in-depth discussion of the clinical implications of the newly established classification framework.

Participants will acquire comprehensive understanding of the most recent CRPS diagnostic criteria, clinical work-up, and novel therapies, allowing for earlier intervention and enhanced patient outcomes.

**At the end of this session, participants will be able to**:

- Illustrate the Valencia update within the context of the CRPS – ICD 11 classification for chronic pain.
- Describe a pragmatic clinical approach to CRPS considering the prevalence of mimicking conditions requiring differential diagnosis.
- Recognize the evidence for emerging therapeutic options such as neuromodulation for CRPS.

**Speaker One**

**Complex Regional Pain Syndrome (CRPS): An Evolutionary Perspective**

Angela Mailis

University of Toronto

This talk will address evolution of the term and concept over hundreds of years from several points of view. Historically the first case of CRPS-like symptoms and signs was described by Ambroise Pare, a 16th century French barber surgeon and personal physician to Charles IX of France. Subsequently descriptions of the condition would appear periodically in the literature from the early 18th century onwards, while it acquired numerous names. A consensus meeting in Orlando, Florida, in 1993 commissioned by IASP produced a new umbrella term, Complex Regional Pain Syndrome or CRPS, as we know it today. Criteria for the diagnosis of CRPS evolved from the 1994 IASP criteria to the Budapest criteria adopted by IASP in 2004 (and validated later), and the recent changes proposed after the Valencia conference in 2019. Concepts about etiology and pathophysiology evolved as well while treatment modalities continue to be variable and questionable in terms of efficacy. The speaker will provide a succinct overview of historical descriptions, nomenclature, epidemiology, diagnostic criteria, pathophysiological concepts and treatment modalities, as background information for the audience.

**At the end of this session, participants will be able to**:

- Illustrate the Budapest Clinical Diagnostic criteria and the Valencia update within the context of the CRPS – ICD 11 classification for chronic pain.
- Enumerate the pathophysiological mechanisms involved in the generation of CRPS and its subtypes.
- Recognize evidence-based treatments for CRPS.

**Speaker Two**

**Pragmatic Clinical Approach to CRPS – Addressing Mimicking Conditions and Differential Diagnosis Challenges**

Nimish Mittal

University of Toronto

Complex Regional Pain Syndrome (CRPS) can be difficult to diagnose because of its clinical heterogeneity and the prevalence of multiple mimicking conditions. CRPS often manifests with symptoms that overlap with other pain syndromes, neuropathies, and musculoskeletal disorders, leading to misdiagnosis and delayed treatment. Improving patient outcomes requires recognizing the need for a systematic approach to differentiate CRPS from its mimicking conditions.

This presentation reviews a pragmatic clinical approach to CRPS, with a particular emphasis on the presentation and clinical characteristics of mimicking conditions that necessitate a meticulous differential diagnosis. This presentation will delineate a comprehensive evaluation strategy comprising a pertinent clinical history, a specific physical examination, and a systematic diagnostic test work up. Key mimicking conditions to be considered include neuropathic pain syndromes, peripheral nerve entrapments, autoimmune diseases, and psychosocial factors. We will discuss the significance of excluding these conditions using distinct clinical characteristics, imaging studies, laboratory tests, and electrodiagnostic assessments. By addressing the difficulties of differential diagnosis and taking into account the prevalence of mimicking conditions, this pragmatic clinical approach aims to enhance early recognition and appropriate management of CRPS, thereby enhancing the quality of life of patients suffering from this mysterious pain disorder.

**Speaker Three**

**Therapeutic Breakthroughs for CRPS**

Anuj Bhatia

University of Toronto

CRPS is a challenging condition to treat. Intravenous infusions of NMDA antagonists and neuromodulation through targeted delivery of electrical stimulation to the spinal cord have provided options to treat patients with refractory CRPS syndromes. Both these treatments are supported by Level 1 evidence and recent advances have resulted in higher success rates with these treatments. However, controversies remain about the appropriate dose of ketamine to treat CRPS, the durability of its effect, and the role of adjuvants. Neuromodulation of the spinal cord has evolved over the last 20 years with several new stimulation waveforms and targets (e.g., dorsal root ganglion) available to enhance success rates.

Dr. Bhatia will discuss patient selection, optimization of outcomes, and adverse effects of these two resource-intensive treatments for CRPS while providing a summary into ongoing research in these fields. He will also share the experience of his team and ongoing research at his Institution in the use of these therapies to treat CRPS.

**Women’s Chronic Pain and Prescription Opioid Use: Implications for Practice and Policy**

**Session Chair:** Nancy Poole

Center of Excellence for Women’s Health

**Session Abstract:** Women report more chronic pain than men and are more likely to be prescribed and rely on opioids for pain management. Yet, women’s experiences with prescription opioid use for chronic pain management, and how these experiences impact policy and practice in the context of healthcare are not well documented. This symposium will bring clinicians, researchers, and people with lived and living experience together to discuss innovative avenues for integrating sex, gender, trauma, and equity-informed considerations into healthcare provision and women’s pain management. The symposium will focus on both sex and gender-based analysis plus (SGBA+) and women’s experiences of chronic pain and prescription opioids. It will also explore how sex and gender related factors affect women’s experiences of pain and prescribed opioids, and interactions with healthcare providers; and seek innovative solutions to bridge these gaps. The speakers will share a range of resources, including recently released reports, clinical network opportunities, digital guides, online learning platforms, infographics, and information sheets that can be used by healthcare providers, educators, policy makers and women with chronic pain themselves, in building capacity and responses to this urgent health issue.

**At the end of this session, participants will be able to**:

- Explore how an innovative case management and mentoring platform on chronic pain and opioids can support service provision and women’s health outcomes.
- Describe the importance of sex and gender science and SGBA+ in our conceptualizations and responses to chronic pain.
- Offer resources for healthcare providers, educators, policymakers, and women with lived and living experience of chronic pain to support women’s chronic pain management.

**Speaker One**

**Applying SGBA+ to the Pain Action Plan: Opportunities to Augment Research and Action**

Lorraine Greaves

Center of Excellence for Women’s Health, University of British Columbia, School of Population and Public Health

The experience of chronic pain is influenced in all diverse groups by sex and gender related factors, as well as economic and cultural issues. Analysis of, and attention to sex-specific conditions and gender-related inequities are needed. Women are more likely to be misdiagnosed with certain conditions, less likely to receive appropriate pain medication, and less likely to be in clinical trials and other research on pain. This presentation will provide an overview of the importance of sex and gender science and sex and gender-based analysis plus (SGBA+) and describe a new report that will augment the work of the Canadian Pain Task Force Report and Action Plan for Pain in Canada, with SGBA+ and equity-related information, analysis, and direction. The report is specifically dedicated to advancing goals of the Action Plan related to an equitable continuum of care, developing sex and gender-informed educational supports, and a sex and gender-informed research agenda through improving knowledge, skills and research in this area. This presentation will assess both content and process recommendations using SGBA+ as the lens to offer granularity and detail to actions, policies and program directions going forward.

**Speaker Two**

**Sex and Gender-Based Analysis of Opioid Prescriptions for Women with Chronic Pain: Patterns, Risks, and Disparities**

Andrea Furlan

University of Toronto

This section of the symposium will provide an examination, grounded in Sex and Gender-Based Analysis (SGBA), of the complex issue of prescribing opioids for women suffering from chronic pain. We shall investigate the utility of the opioid risk tool, discerning its capacity to discern distinct risk profiles for opioid addiction between the male and female populations. We will consider how this tool and sex/gender-informed approaches to opioid prescribing have been considered in Project ECHO (Extension for Community Health-care Outcomes) an innovative, telementoring program that amplifies the capacity of health-care providers to deliver best practice care to the underserved in their own communities. The focal objective of this section of the symposium is to contribute to the evolving landscape of clinical practice guidelines by highlighting the imperative need for a comprehensive consideration of risk assessment, prescribing protocols, maximum dosage recommendations, and ongoing monitoring specific to women receiving opioid therapy.

**Speaker Three**

**Moving Past the Prescription: Addressing Women’s Complex Needs for Pain Management**

Lindsay Wolfson

Center of Excellence for Women’s Health

This presentation will describe sex, gender, trauma, and equity-informed resources developed for healthcare providers, educators, policymakers, and women with experience of chronic pain. The findings will be drawn from a two-year project led by the Center of Excellence for Women’s Health and funded by Health Canada’s Substance Use and Addiction Program. This project translated women’s lived and living experiences of chronic pain and prescription opioid use into resources for health care providers. The project, led by a person with chronic pain, wove findings from literature reviews on 1) women’s experiences with prescription opioids for chronic pain and 2) effective pain management strategies for women, informed by women with chronic pain, to develop responsive resources.

The findings reinforced the role of prescribed opioids on women’s quality of life, and how differences in care were related to social factors such as race, class, age, sexual orientation, chronic condition, geography, self-advocacy, access adequate care, and stigma associated with the opioid crisis.

The developed resources – inclusive of a digital guide, infographics, information sheets, and discussion guides – respond to women’s emphasized need for healthcare providers and policymakers to have a better understanding of women’s experiences of chronic pain, the benefits and harms of opioid use for women who live with chronic pain, and the need for comprehensive pain management plans. This presentation will provide an overview of the resources, how they can be used by a range of practitioners, and the ways in which women’s voices remained at the center of their creation.

